# Is a Positive Relationship Between Fertility and Economic Development Emerging at the Sub-National Regional Level? Theoretical Considerations and Evidence from Europe

**DOI:** 10.1007/s10680-018-9485-1

**Published:** 2018-05-08

**Authors:** Jonathan Fox, Sebastian Klüsener, Mikko Myrskylä

**Affiliations:** 1 John F. Kennedy Institute for North American Studies, Freie Universität Berlin, Lansstr. 7-9, 14195 Berlin, Germany; 2 Max Planck Institute for Demographic Research, Konrad-Zuse-Str. 1, 18057 Rostock, Germany; 3 Vytautas Magnus University, Kaunas, Lithuania; 4 London School of Economics, London, UK; 5 University of Helsinki, Helsinki, Finland

**Keywords:** Fertility, Income, Economic development, Sub-national regions, Regional variation, Europe

## Abstract

Evidence for nation-states suggests that the long-standing negative relationship between fertility and economic development might turn positive at high levels of development. The robustness of the reversal continues to be debated. We add to this discussion from a novel angle by considering whether such a reversal could also occur at the sub-national level within highly developed countries. Our contributions are both theoretical and empirical. We first discuss important trends which might foster the emergence of a positive fertility–development relationship across regions of highly developed countries. These include shifts in family policies, changes in the spatial organisation of the economic sphere, and selective international and internal migration processes. In order to explore whether we observe tendencies towards a reversal, we investigate data covering 20 European countries subdivided in 256 regions between 1990 and 2012. We document a weakening of the negative relationship between fertility and economic development within many countries, and among some countries the emergence of a positive relationship. These findings do not seem to be driven by postponement effects alone. However, there is substantial variation in the fertility and the economic development levels at which such tendencies towards a reversal are observed.

**Electronic supplementary material:**

The online version of this article (10.1007/s10680-018-9485-1) contains supplementary material, which is available to authorized users.

## The Negative Association Between Fertility and Economic Development—Is it Here to Stay?

Dominant demographic theories such as the demographic transition and second demographic transition frameworks postulate a negative relationship between development and fertility. As societies progress, fertility tends to decrease (Kirk 1996; Lesthaeghe 2010). Proposed potential mechanisms include the opportunity costs of childbearing, investments in the quality instead of the quantity of children (Becker 1960), or in the case of the second demographic transition, rising higher-order needs conflicting with childbearing aspirations (Lesthaeghe 2010). These frameworks fit the available evidence over most of the twentieth century, as areas with advanced levels of economic development tended to have lower fertility levels than less developed areas. Perhaps due to the implicit assumption of demographic transition theory that fertility decline is not reversible (Burger and DeLong 2016), attention has accordingly been directed towards the implications of this relationship, particularly with regard to population age structures (e.g. Wigglesworth 2016; Zeihan 2014). Recently however, new theoretical considerations have been presented which suggest that at high levels of development the relationship between development and fertility might turn positive (Esping-Andersen and Billari 2015; Goldscheider et al. 2015). These new theories point to improvements in gender equality as potential driver of rising fertility levels in highly developed countries.

Concurrent with these theoretical shifts are shifts in the empirical evidence. While a global national-level comparison still tends to demonstrate a negative relationship between fertility and development levels, recent evidence suggests that among high-income countries this relationship may transition from negative to positive. Myrskylä et al. (2009) found that at high levels of socioeconomic development, as measured by the Human Development Index (HDI), the relationship between development and fertility turns from negative to positive. This observation has been referred to under different names in the literature, including “the income/development-fertility reversal”, “inverse J-shaped association”, or “convex relationship between income/development and fertility”. These terms are used synonymously throughout this paper. Luci-Greulich and Thévenon (2014) also determined a convex relationship between GDP per capita and fertility among OECD countries. In line with the above-mentioned theoretical considerations on shifts in gender relations, the authors attribute the new patterns in the fertility–development association to the changing relationship between female employment and fertility. Myrskylä et al. (2011) also argue that a high level of gender equality is critical for the association between development and fertility to reverse.

However, whether such a reversal is occurring remains contested. Ryabov (2015) applied factor analyses with cross-sectional US county data to state that development and fertility are negatively related in the USA. Harttgen and Vollmer (2014) used a revised HDI index and its health, education, and standard of living components to argue that the finding of a positive association between development and fertility in Myrskylä et al. (2009) is sensitive to the choice of the development measure. A more general criticism accepts the presence of a “reversal” but questions whether it is occurring due to increases in the quantum of fertility, or whether it is simply a sign of the end of a period characterised by strong fertility postponement. Period fertility rates can be depressed as a result of women postponing their births to higher ages, and when fertility postponement slows down, period fertility may increase (Bongaarts and Sobotka 2012; Goldstein et al. 2009). Bongaarts and Sobotka (2012), for instance, argue that recent rises in fertility in highly developed countries are mostly driven by postponement effects. They suggest that a reversal of the association between development and fertility is driven primarily by further advances in economic development and the simultaneous slowing or ending of fertility postponement. These two processes may simply happen simultaneously without a causal relationship between the two. However, analyses of cohort fertility trends suggest that at least a portion of the rising fertility rates in highly developed countries is attributable to true increases in the quantum of fertility (Myrskylä et al. 2013; Schmertmann et al. 2014). Furthermore, even if postponement effects are an important mechanism that links trends in economic development to changes in fertility, the relationship between economic development and fertility remains important.

The goals of this paper are to add the sub-national regional dimension to this discussion both in terms of theoretical considerations and empirical findings, and to explore to what extent changes in postponement contribute to the observed patterns in European countries. Within European countries, sub-national regions vary in their levels of income/development and fertility, which can be adequately captured with cross-country comparative data. Relative to cross-country differences, regions within countries tend to be generally more similar in their cultural and policy frameworks (Basten et al. 2011; Decroly and Grasland 1993; Watkins 1991). As such, exploiting variation in the sub-national dimension offers potential to improve our understanding of the fertility–income relationship. Findings for nation-states could be driven by national-level variation in economic structures and policies simultaneously affecting both fertility and levels of income or development. Through the addition of the sub-national level, these national-level trends can be controlled for, as well as any regional-level differences that do not change over the period of study or exhibit regional spatial autocorrelation.

In our theoretical section, we discuss recent developments that might attenuate or reverse the long-standing negative fertility–income relationship across sub-national regions in highly developed countries. To our knowledge, this paper represents the first attempt to develop a theoretical argument why we might witness a turnaround in the fertility–income relationship across sub-national regions. In the subsequent empirical analysis we explore whether we can detect tendencies towards a turnaround of the fertility–income relationship across regions within countries. Our analysis focuses on Europe, as previous work has shown that a number of highly developed European countries appear to be susceptible to a reversal in the fertility–income relationship (Luci-Greulich and Thévenon 2014; Myrskylä et al. 2009, 2011). Specifically, we use data for 20 European countries divided in 256 sub-national regions over the period 1990–2012.^1^ Our outcomes provide evidence for attenuation and tendencies towards a reversal from negative to positive in the relationship between income and fertility in a number of European countries. While postponement effects do appear to contribute to the observed trends, the estimated positive association at high levels of development is generally robust to attempts to control for postponement. Furthermore, across countries there is substantial variation in the fertility and economic development levels at which such tendencies towards an attenuation or reversal are observed.

## Development, Economic Centres and Peripheries, and Fertility—Theoretical Background

### The Emergence of the Negative Relationship

A vast literature has documented the negative relationship between income or development and fertility (e.g. Becker 1960; Cleland and Wilson 1987; Goldstein and Klüsener 2014; Guinnane 2011; Oppenheim Mason 1997). We will thus primarily focus on aspects relevant for understanding variation within countries, as this dimension has traditionally received less focus in research on the relationship between income and fertility. As we find within many countries a positive relationship between per capita income levels and the degree of urbanisation, we will also cover considerations related to urban–rural and centre–periphery disparities.^2^

Europe’s distant past has been arguably characterised by Malthusian cycles of boom and bust in which increases in income generated increases in fertility (Guinnane 2011). Over the course of the demographic transition during the nineteenth and twentieth centuries, this relationship began to break down, such that higher levels of per capita income no longer translated into higher levels of fertility (Dribe and Scalone 2014; Skirbekk 2008). This was the case for both urban and rural areas (Klüsener et al. 2014). One explanation for why such a transition may have occurred was offered by Becker (1960), who considered the ability of parents to trade investments in the quantity of children for investments in quality (see also Lawson and Mace 2011). The incentive to invest in quality over quantity can be amplified by enhanced social mobility opportunities, which have mostly emerged in centres of economic activity (Lipset and Bendix 1991). This likely increased the propensity that shifts from quantity to quality occurred first in highly developed areas, and this may have contributed to the negative association between economic development and fertility across regions within European countries.

A related process during this period was the rise in the financial and opportunity costs of having children. In high developed areas costs of living for families increased faster than in less developed areas. In parallel, there were also substantial changes in the spatial organisation of the economic sphere (Hayford 1974). Prior to the industrial revolution, agricultural labourers usually lived close to their fields, and proto-industrial forms of work such as weaving or craftsmanship often occurred in the household. The shift of employment to factories and commercial zones increased especially among employees in highly developed urban areas the likeliness to be away from home for a substantial part of the day. Many of these new employment opportunities also absorbed female workers, who had previously been under-utilised in formal labour markets (Blythell 1993; Kocka 1990). Taking up such employments limited the time available for childrearing tasks. Overall, these financial and opportunity costs also fostered a negative relationship between economic development and fertility levels at the sub-national level.

### Why We Might Witness a Reversal of the Relationship

In discussing the potential role of shifts in family policies, we first look at past patterns from which these shifts represent a deviation. Many European countries faced strong fertility declines in the first half of the twentieth century, which led governments to enact pro-family policies that mostly provided child benefits or tax deductions and were targeted towards a male-breadwinner model (Gauthier 2002; Lorimer 1945; PERFAR 2017). Apart from additional support given to families with multiple children, these payments were usually not means-tested (PERFAR 2017; see also Gauthier 2002). As these benefits were imperfectly adjusted for costs of living, in real terms they were most beneficial for residents of less developed regions within a country. Thus, if this first generation of family policies affected fertility, it would likely have intensified the negative relationship between economic development and fertility levels across regions within countries.

In recent decades, the focus of family policy reforms has shifted to extending parental leave schemes and childcare (Gauthier 2002; PERFAR 2017). Rather than providing transfer payments, these new family policies are aimed at decreasing the opportunity costs of childrearing (Gauthier 2007). Such costs are particularly high when both partners have acquired substantial human capital and have high earning potential. Parental leave support payments are often linked to prior income levels, which better adjusts for individual and regional variation in cost of living. In addition, because many parental leave programs guarantee that after taking a period of leave a parent can return to his or her previous job, uncertainties regarding future career options are reduced. The improved access to publicly supported childcare decreases the time trade-off parents would face without such services, so mitigates parental opportunity costs. Overall, relative to the first generation of family policies, this second generation likely provides better support for dual-earner couples with high incomes. Since these individuals are concentrated in highly developed areas (de Meester and van Ham 2009), we believe these policies to weaken the negative relationship between income and fertility, or even to contribute to a turnaround.

Changes in family policies also support recent changes in gender roles towards a higher degree of gender egalitarianism (Neyer and Andersson 2008). Esping-Andersen and Billari (2015) argue that fertility tends to decline when women enter the labour market in large numbers, but that it begins to increase if a society approaches gender egalitarianism. Family policies that support parents in reconciling work and family can be a crucial element in this process. Since highly educated women are more likely to see gender egalitarianism in their partnerships than their less educated counterparts (Esping-Andersen and Billari 2015), and since these women are more concentrated in highly developed regions, this shift in gender roles could also more positively affect fertility in these regions.

For this second wave of family policies, there is temporal variation across European countries in the introduction of a family policy mix that supports parents in reconciling family and career goals. Northern and Western European countries were the forerunners in implementing such policies, while Central-Western, Eastern, and Southern European countries lagged behind (Gauthier 2002; PERFAR 2017; Thévenon 2011). Especially in Southern Europe, policies continue to be fragmented and thus do less to mitigate the uncertainty over whether both partners would be able to reconcile family and career goals after the birth of a(nother) child (Thévenon 2011). This variation might also help to explain why Northern and Western European countries are more likely than Central-Western and Southern European countries to have experienced a stabilisation of cohort fertility at rather high fertility levels (Myrskylä et al. 2013; for empirical support for a link between family policies and fertility trends, see, e.g. Kalwij 2010 and Klüsener et al. 2013).

In addition to these shifts in family policies, another important process influencing recent trends are changes in the spatial organisation of the economic sphere. The Internet and associated technologies have opened up opportunities for individuals to create more flexible working arrangements than previously possible. Under such arrangements, employees are freed from the obligation to be present at a regular workplace for all work hours (see also Gauthier 2007). Just as industrialisation changed the spatial organisation of economic activities in the nineteenth and twentieth centuries, technological advancements are changing the spatial organisation of economic activities in the twenty-first century. In this process, residential households are regaining importance as places for generating income. Flexible working arrangements offer new options for combining childrearing obligations with career plans, and as such also support increased gender equality. This could have positive effects on the fertility decisions of working couples, as childbearing intentions are affected by the amount of subjective control over his or her work a person is able to achieve (Begall and Mills 2011). Although the share of employees able to make use of such flexible opportunities is still low in many countries, these opportunities have increased in recent years, especially for the highly educated (e.g. Beninger and Carter 2013). As long as centrality and agglomeration effects will remain relevant in economic activities (Krugman 1998), these highly educated will be concentrated in more economically advanced areas. Thus, we believe that increases in flexible work arrangements offer potential to contribute to an attenuation or reversal of the sub-national regional-level relationship between income and fertility.

The final important trend related to the potential reversal between fertility and income across sub-national regions is that of selective international and internal migration and the ensuing developmental challenges in peripheral areas. With the industrial revolution, the relevance of land as a production factor declined. This development, together with innovations in agricultural production that decreased the demand for rural labour, contributed to substantial migration streams from peripheral to more centralised areas. As a general trend, this phenomenon continues today. Migration is usually selective in terms of individual assets, such as health status and the resources to migrate (Abramitzky et al. 2012; Blau and Mackie 2017; Mehta and Elo 2012). As such, this long period of outflow from less developed regions might create development challenges in these areas, with negative effects on available human capital, partner markets, and fertility levels.

Effects of migration on fertility patterns can be observed not only within countries, but also between countries. Like domestic migrants, international migrants frequently tend to settle in highly developed areas, as these are usually the places with more opportunities and social and communication links to countries abroad (Bonifazi et al. 2008). The inflow of international migrants into highly developed areas can have significant positive effects on fertility levels, particularly in low-fertility contexts (Billari and Dalla-Zuanna 2011). This can be both due to quantum and tempo effects, as many foreign migrants postpone fertility until they reach their destination (Wilson 2013). Overall, processes of selective international and internal migration might be of particular relevance for countries with low fertility levels. If these migration processes contribute to a positive association between economic development and fertility, they may do so at fertility levels far below the replacement level.

### The “Noise” of Mid-Term Transitions Within Long-Term Transitions

The broad trends outlined above are long-term transitions that operate over decades or even centuries. In addition to these long-term developments, there are also transitions that operate at shorter time intervals. These mid-term transitions might cause temporary shifts in the fertility–development relationship at the sub-national level, challenging efforts to monitor long-term shifts in this relationship. Two shorter-term transitions discussed here are the postponement of births to higher ages and the transition processes in Eastern Europe following the collapse of communism.

As mentioned above, one interpretation of the empirical evidence pointing to a turnaround in the relationship between period fertility rates and income is the end of postponement (Bongaarts and Sobotka 2012). The reversal in the association between development and fertility from negative to positive can at least in part be linked to a temporary depression of fertility levels due to the postponement of births, and the following recovery when postponement ends. If highly developed regions within countries experience postponement earlier or to a greater degree, this might create temporary distortions in the association between development and fertility at the sub-national level. However, postponement effects on fertility should not be interpreted as resulting from demographic pressures alone. Social and economic forces start the process of fertility postponement, and it is likely that these forces play an important role in determining how long postponement continues. In addition, recent empirical evidence of stabilising or even increasing cohort fertility rates in a number of European countries (Myrskylä and Goldstein 2013; Myrskylä et al. 2013; Schmertmann et al. 2014) suggests that also upward trends in the quantum of fertility might play a role.

The post-communist transition crisis that many Eastern Europeans experienced during the 1990s is another shorter-term transition that may affect our ability to study long-term shifts in the sub-national level relationship between fertility and economic development. The economic crises of differing levels across these countries had consequences for both postponement decisions and quantum fertility levels (Myrskylä et al. 2013; Sobotka 2003). Exploratory analyses of our data suggest that regions with varying income levels were not substantially different in their initial fertility reaction to the crisis, but that high developed areas seemed to have generally recovered more quickly (see also Alam et al. 2005 and Macours and Swinnen 2008). These uneven recoveries from the transition shock might appear as the emergence of a positive relationship, even though they are likely “noise” of a distortion due to the post-communist crisis.

As fertility postponement and the post-communist transition are highly relevant for our study period (1990–2012), it is important to consider these aspects in our analyses of changes in the fertility–income relationship. To explore how postponement affects our analysis, we compare models using the total fertility rate (TFR) as a fertility measure with models in which the TFR is replaced by a tempo-adjusted TFR. To account for the potential distortion effects due to the post-communist transition, we separate the sample between Western European countries and the former communist Eastern European countries. As a final comment, the 2007 economic recession may also represent a short-term distortion. We explore this further in the online appendix of consistency checks.

## Data

In this section, we briefly outline the data and variables used. For a detailed discussion of the data, including summary statistics and our decisions on corrections, we refer readers to the “Data Appendix”. Our analysis relies on fertility and economic data at the NUTS 2 level, where NUTS is a hierarchical classification system of the European Union (EU) that distinguishes countries and sub-national regions of comparable population size across Europe. We analyse NUTS 2 level data as this is the lowest level of division for which the required fertility and economic data are available. NUTS 2 regions generally have populations between about 800,000 and three million inhabitants. To explore whether the association between income and fertility turns from negative to positive at high levels of development we require two variables (per capita income and per capita income squared) that reflect the variation within countries; thus, at least three different regions are needed to make an estimate. To ensure at least one degree of freedom, we omit all EU/EFTA (European Free Trade Association) countries with less than four regions from the analysis. Switzerland is also excluded due to data limitations (for additional details see “Data Appendix”).

To calculate the TFR and an estimate of a tempo-adjusted TFR, age-specific birth count and population data were drawn from a combination of sources. Most of the data were obtained through Eurostat. However, there are some large gaps in the time series for a number of countries during the period of observation. We thus made substantial efforts to fill in these gaps using data directly derived from national statistical offices. This also allowed us to correct some inconsistencies in the Eurostat data (see “Data Appendix”). For some countries, it was unfortunately not possible to obtain a complete time series of sub-national fertility rates since 1990. We face some variation in the quality of the fertility data, which is mostly driven by challenges statistical offices faced in accounting for changes in the population due to under-registered migration during intercensal periods. This aspect is especially important for Eastern European countries which witnessed significant outmigration in the early 1990s and after the 2004 and 2007 EU enlargement rounds. Most of the population data used in our analyses has been retrospectively adjusted by the statistical offices based on the outcomes of the 2001 and 2011 census rounds. Nevertheless, we encourage caution when interpreting the Eastern European results. This is particularly true for models which include the not-backward-adjusted Romanian data between the censuses of 2002 and 2011 (see below and “Data Appendix”).

Economic data were obtained from Cambridge Econometrics (CE). These data primarily originate from Eurostat and were adjusted by CE to 2005 Euros using purchasing power parity deflators. Employee compensation per capita is chosen as our measure of per capita income, as it accounts for household income levels better than the more commonly used Gross Domestic Product (GDP). GDP, which is calculated in the dataset as Total Gross Value Added, plus taxes and less subsidies, captures total income generated by both firms and households. This provides a good measure to compare the overall economic development of different areas. However, as we are interested in variation in private household incomes across regions, it is potentially a challenge that GDP comprises both labour and capital income. Capital owners may be less likely to reside in the same region relative to labourers, so information on capital income may be less relevant for fertility decisions in the region. For that reason we focus on the aforementioned employee compensation, which consists of wages and salaries, and of employers’ social contributions. This measure of income should more closely approximate the labour income earned by individuals in the different regions. Employment participation rates and the demographic structure of the population can vary across our regions. We focus, however, on employee compensation per capita instead of compensation per worker or per “active population” member (which includes both employed and unemployed people, but not economically inactive, such as (pre-)school children, students, and pensioners) to gauge the experienced economic well-being of the population.^3^

We acknowledge that employee compensation per capita may miss income generated by households in other ways. If regions differ significantly in how that income is generated, employee compensation may reflect a distorted measure of actual household income levels. Sub-national regions might, for example, vary in the amount of remittances received. In this case, employee compensation per capita will overestimate household incomes in sending regions and underestimate household incomes in receiving regions. If remittances are important for the relationship, then using GDP per capita as a measure of economic development that includes received remittances spent locally should result in significantly different model estimates. That we do not observe substantial differences between models using GDP per capita and employee compensation per capita indicates that these distortions do not likely represent a significant source of bias.

## Empirical Analyses

### Cross-Sectional Comparisons

**Fig. 1:**
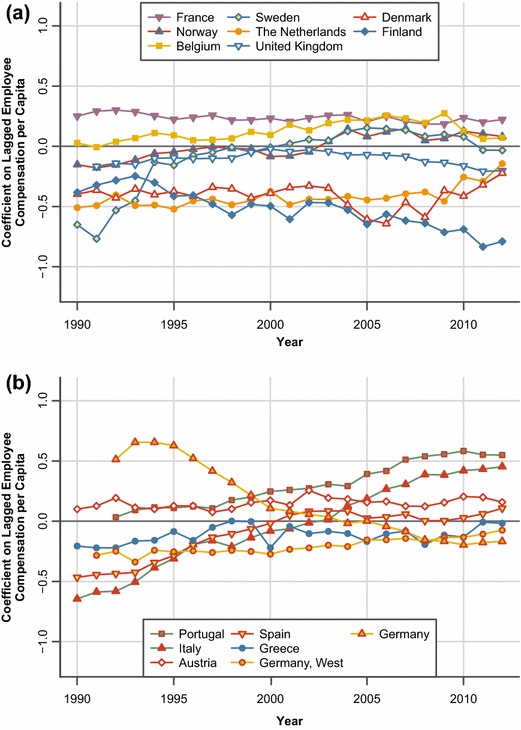
Predicted changes in total fertility rates from a 1% change in income: Western European countries, 1990–2012. **a** North and West, **b** Centre and South. Predicted fertility changes are calculated annually from cross-sectional country-specific regressions. Each data point then represents the coefficient of the lagged natural log of employee compensation per capita regressed on the TFR annually across regions for each of the different countries. Employee compensation is defined as the total remuneration, in cash or in kind, payable by an employer to an employee in return for work done by the latter. It consists of wages and salaries, and of employers’ social contributions and is adjusted to 2005 Euros. *Source*: Eurostat, Statistical Offices, Cambridge Econometrics; own calculations

**Fig. 2:**
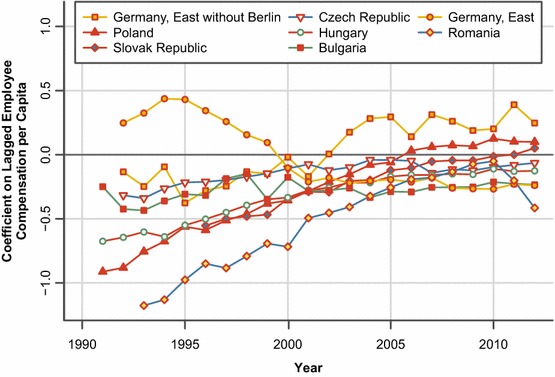
Predicted changes in total fertility rates from a 1% change in income: Eastern European countries, 1990–2012. Predicted fertility changes are calculated annually from cross-sectional country-specific regressions. Each data point then represents the coefficient of the lagged natural log of employee compensation per capita regressed on the TFR annually across regions for each of the different countries. Employee compensation is defined as the total remuneration, in cash or in kind, payable by an employer to an employee in return for work done by the latter. It consists of wages and salaries, and of employers’ social contributions and is adjusted to 2005 Euros. “Germany, East” includes former West Berlin as part of the city of Berlin. *Source*: Eurostat, Statistical Offices, Cambridge Econometrics; own calculations

As a first step of analysis, we look at trends in the cross-sectional relationship between income and fertility levels across NUTS 2 regions for each of the sample countries between 1990 and 2012. We do this by performing cross-sectional regressions on a by-country annual basis. Thus, for each year and for each country, TFRs are regressed on the lag of adjusted employee compensation per capita. Figures 1 and 2 plot the country-specific cross-sectional correlations for countries in Western Europe (Fig. 1) and Eastern Europe (Fig. 2). For readability, Fig. 1 is further subdivided into those Western European countries situated in the north and west of Europe (Fig. 1a) and those situated in the centre and the south (Fig. 1b).

From Fig. 1a, we see that in the early 1990s the relationship was negative for almost all displayed Western European countries in the north and west. The only exceptions were France and Belgium. For Finland, the cross-sectional correlation remained consistently negative throughout the study period, and became slightly more negative in the most recent years. However, apart from Finland, we only observe for France and the UK a more negative correlation coefficient in 2012 than in the early 1990s. Among the five countries that experienced increases, Norway and Sweden stand out. Both countries moved from having a relatively negative correlation to a positive one. However, for Sweden, the correlation declined in the last 2 years of the period to a value just below zero. Additionally, although the correlation has remained negative for both Denmark and the Netherlands, these two countries have recently been experiencing movement towards reporting a less negative relationship between per capita income and fertility.

If we turn to the Western European countries in the centre and the south of Europe (Fig. 1b), one trend that stands out is the big change in the fertility–income relationship for Germany from high positive to negative values. The high positive values in the early 1990s were related to the depression in fertility in the eastern German regions as a result of the post-communist transition. At that time, the more affluent regions in western Germany also had relatively low fertility levels, but these were still higher than those of eastern Germany. Eastern Germany still lags behind western Germany in terms of economic development, but fertility has generally recovered and is currently higher than in western Germany (Goldstein and Kreyenfeld 2011). As a result, the coefficient for Germany turned negative in the 2000s. Because the effects of the previous division of Germany may linger, we derive the coefficients for the western and the eastern German areas separately. The trend for western Germany is included in Fig. 1b, while the trend for eastern Germany is grouped with those of the other former communist countries in Fig. 2. As visible in Fig. 1b, nearly all of the countries had a negative correlation coefficient in the early 1990s, with Austria and Portugal being the exceptions. Apart from (united) Germany with its explainable concave trend, all of the displayed countries show a tendency towards an increasing coefficient. As a result, we estimate a (slightly) negative association between income and fertility at the end of the study period for western Germany and Greece only, while Italy and Spain have joined Austria and Portugal in reporting non-negative coefficients.

Figure 2 presents the trends for Eastern Europe. As the countries in Fig. 1b, the Eastern European countries also display a generally consistent pattern. Only eastern Germany had a positive coefficient at the beginning of the study period, which is likely an artefact of the data. Approximately, two-thirds of the population of Berlin are living in former West Berlin, which had been part of West Germany during the division of the country. As a result, Berlin was less affected by the drastic post-communist transition crisis eastern Germany experienced in the early 1990s. Thus, despite having relatively low fertility levels, Berlin was the region of eastern Germany that had the highest economic development and fertility levels in the 1990s. This pattern changed as the crises passed, with the fertility levels of many eastern German regions overtaking those of Berlin. If Berlin is omitted from eastern Germany, we also obtain a negative coefficient at the beginning of the study period.

The negative relationship observed in all Eastern European countries in the early 1990s weakened throughout the 1990s and 2000s, so that we witness for most of these countries fertility–income correlations of around zero in 2012. Only Romania experienced something of a reversion in the final years of our study period. However, this seems to be an artefact of the shortcomings in the Romanian population estimates for the 2000s that created a downward bias in fertility rates in less developed regions and an upward bias in the more developed ones (see “Data Appendix” for more details). This bias might have substantially contributed to the observed “reduction” in the negative coefficient in the 2000s, as the coefficients for the years following the 2002 and 2011 census years are not far apart. While other Eastern European countries also face challenges with their population exposures, it is to some degree reassuring that we do not observe breaks in the time trends of the cross-sectional coefficients for any other country in Figs. 1 and 2 around the different census years (for country-specific census years, see the “Data Appendix”). As to the potential impact of the 2007 crisis, we note that we do not witness massive changes in the trends of our cross-sectional coefficients during the recession period (see Figs. 1 and 2).

**Fig. 3:**
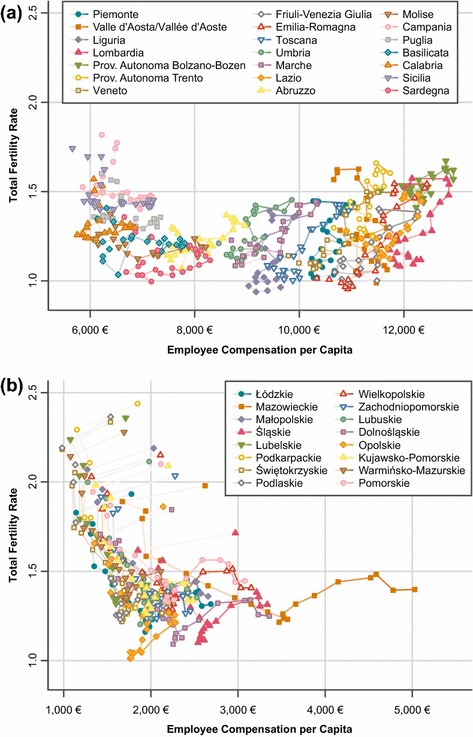

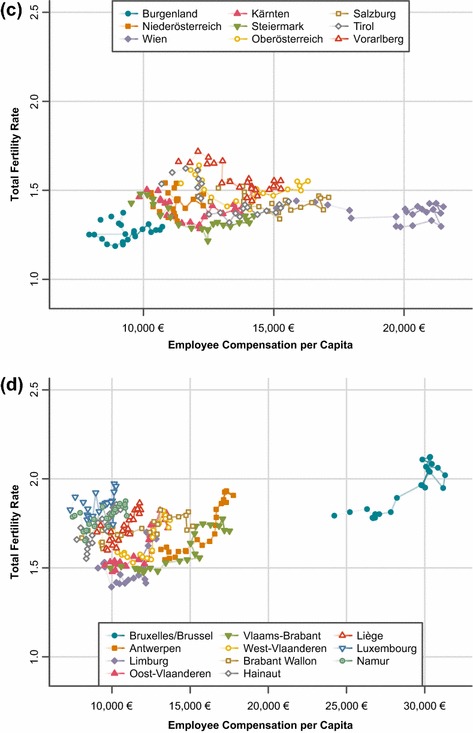
Regional trajectories by fertility and income within four selected countries, 1990–2012. **a** Italy, **b** Poland, **c** Austria, **d** Belgium. *Source*: Eurostat, Statistical Offices, Cambridge Econometrics; own calculations

Overall, the trends of these cross-sectional associations provide support that over the last two decades most European countries have experienced a lessening of the negative association between economic development and fertility across their regions. In many countries, this association has even turned positive. However, the fertility and economic levels at which countries report such tendencies vary substantially across countries. To illustrate this, we show in Fig. 3 the trajectories of sub-national regions by fertility and income for a selected number of countries. In Italy (Panel a), Poland (Panel b), and Austria (Panel c), the tendencies towards an attenuation or reversal occur at a TFR of around 1.3. In Belgium (Panel d), on the other hand, we can witness this shift at a higher TFR level of 1.7. Furthermore, the employee compensation levels at which this trend pattern is observed vary substantially across countries (from 2000 Euros in Poland to more than 10,000 Euros in Austria and Belgium).

Figure 3 also allows a look into the trends during the last years of the observation period, when many countries were affected by the 2007 recession. As the trends evolve over time, the lines are plotted in more saturated colours allowing the identification of the final years in the panel (2008–2012), when many countries were affected by the recession. In Italy especially, many regions exhibited a backwards bend in their income trends during the recession. This was usually paralleled by downward shifts in fertility, indicating a positive association between fertility and income during the crisis period.

### Country-By-Year Fixed Effects

The preceding analysis looked at the evolution of the cross-sectional associations between income and fertility levels over time within different European countries. In this second part of our empirical section, we investigate how changes in income are related to changes in fertility by applying country-by-year fixed effects models. We emphasise these models are not specified to identify causal effects of income on fertility, but to provide a broad perspective on the association between these two variables across European regions. With that goal in mind, the models below include the TFR or the tempo-adjusted TFR as dependent variable, and as explanatory variables, the prior year log of regional income and its square (as measured by employee compensation per capita). The square term is included to test for the convexity of the fertility–income relationship. For unobserved factors affecting income and fertility jointly (e.g. family policies and cultural norms), we attempt to control for with the fixed effects.

The country-by-year fixed effects model is based on the assumption that regions within a country are identical in those factors related to both income and fertility. We justify this assumption with the observation that, for Europe, the national level is most relevant for aspects that might affect the general levels of income and fertility in a country (Basten et al. 2011; Decroly and Grasland 1993; Watkins 1991). This includes standardised education curricula, national mass media, migration policies, and policies affecting economic growth, work-family reconciliation, and gender equality. We acknowledge that we might also face unobserved heterogeneity at the sub-national level, especially in countries that are highly federalised (see, for instance, Baizan 2009 on Spain). As such, our online appendix includes consistency checks with regional fixed effects models (controlling for time-invariant unobserved effects at the NUTS 2 regional level) and spatial panel models that control for spatial autocorrelation in the error term among neighbouring regions. Section 4.4 below summarises the findings from these and other consistency checks presented in the online appendix.

In notation form, our main model is specified as follows:

1 $$ y_{i,c,t} = \chi_{c} *\lambda_{t} + \beta_{1} \ln \left( {inc_{i,c,t - 1} } \right) + \beta_{2} \ln \left( {inc_{i,c,t - 1} } \right)^{2} + \varepsilon_{i,c,t} $$

with *y*_*i*,*c*,*t*_ denoting the outcome variable (TFR, tempo-adjusted TFR) at time *t* (measured in years), in country *c*, and in region *i*. The first term, *χ*_*c*_ * *λ*_*t*_, controls for the country-by-year fixed effects. The inclusion of this interaction term accounts for a country-specific effect that varies over time. This is in contrast to the inclusion of country and year fixed effects, which would control for the time-constant country-level factors and the time-varying factors common to all of the countries in the panel. Income per capita is represented by *inc* and appears in both the linear and the squared terms. The error term *ɛ*_*i*,*c*,*t*_ is assumed with conditional mean zero. We estimate this model separately for the full sample, for regions part of Western European countries (including former West Germany), and for regions part of the Eastern European countries (including former East Germany).^4^ Germany is divided into its former East and West German regions since combining them, as seen in Fig. 1a, likely violates the assumption of NUTS 2 level similarity within the country-by-year fixed effects models.

Estimates from the model using TFR are presented in Table 1, as is the associated level of per capita income at which the results indicate any sort of a fertility reversal. There is a great deal of statistical uncertainty regarding the specific income level at which a reversal might occur. So we interpret this inversion point simply as a measure of the magnitude of the coefficients, not as the specific income level at which the association between income and fertility is expected to turn positive. The model results for the TFR indicate in point estimate form what was visually apparent in Figs. 1 and 2. Estimates from the full sample presented in column 1 indicate a negative association between per capita income and fertility at low levels of per capita income. This diminishes as per capita income increases, and the models that we specified show a positive association at about 19,000 2005 Euros for the combined European sample. Compensation per capita is generally about 40–60% of GDP per capita for the set of regions in the sample. Taking this into account, our outcomes are consistent with the findings that Luci-Greulich and Thévenon (2014) derived based on GDP data. When we compare the Western and the Eastern European countries, the outcomes are generally the same, but the estimates of the inversion point differ. For the Eastern European countries, it occurs at an employee compensation per capita of about 7000 2005 Euros, while for the Western European countries the inversion point is estimated at 24,000 2005 Euros.

**Table 1:** Country-by-year fixed effects, total fertility rate (TFR). *Source*: Eurostat, Statistical Offices, Cambridge Econometrics; own calculations

Independent variables	Europe (combined sample)	Western Europe	Eastern Europe
	(1)	(2)	(3)
Prior year compensation per capita (natural log)	− 1.255 (0.089)**	− 0.884 (0.224)**	− 2.271 (0.184)**
Prior year compensation per capita squared (natural log)	0.064 (0.005)**	0.044 (0.012)**	0.129 (0.011)**
Constant	7.675 (0.403)**	5.957 (1.047)**	11.230 (0.745)**
Inversion point (in 2005 Euros)	18,827 €	23,656 €	6680 €
Country-year interacted fixed effects	Yes	Yes	Yes
Number of observations	5703	4522	1181
Regions	256	200	56
Adjusted R-squared	0.825	0.804	0.835

Robust standard errors in parentheses

Employee compensation is defined as the total remuneration, in cash or in kind, payable by an employer to an employee in return for work done by the latter. It consists of wages and salaries, and of employers’ social contributions and is adjusted to 2005 Euros. Inversion points are calculated using coefficients rounded to the 8th decimal point

**p < 0.01, *p < 0.05, ^+^*p* < 0.1

### Controlling for Tempo Effects in Fertility Trends Across Regions

To examine the extent to which fertility postponement may be contributing to the above results, we attempt to control for tempo effects within the different countries. The arguably most sophisticated approach suggested in the literature requires parity-specific fertility by age for the different regions (Bongaarts and Sobotka 2012). As these data are not available, we turn to a second-best method by estimating the tempo-adjusted TFR using the information on the average age at childbearing. This method has been described and evaluated by Potančoková et al. (2008).^5^

According to Potančoková et al. (2008), the quality of the less sophisticated estimate is high only if the fertility quantum is relatively low, there are no dramatic changes in higher-order fertility, and when the changes in the mean age at childbirth (MACB) are uniform and sufficiently small.^6^ During our study period, higher-order births were not a primary component of the changes in fertility across our European countries. However, in the mean age at childbirth, we observe in some regions in the early stages of postponement changes that exceeded the maximum suggested figure of about 0.1 years per year (Potančoková et al. 2008). This can cause an overestimation of the tempo effect and too high estimates of the tempo-adjusted TFR. Such error in measurement should decrease the likelihood of observing an upturn in the tempo-adjusted TFR in later stages of the postponement transition. Since temporal variance in annual changes introduces a significant amount of noise in the estimates, a smoothed tempo-adjusted TFR ($\widehat{TFR}^{*}$) is used as the dependent variable in Eq. (1).^7^

Comparing the model estimates for the tempo-adjusted TFR in Table 2 with those in Table 1, the results appear generally the same. One difference between Tables 1 and 2 is visible in the coefficients for Eastern Europe. Although they are still highly statistically significant, the magnitude of both the linear and the squared term coefficients have been reduced by about 20–25%, which implies that the increase in fertility that is associated with growing income is smaller when tempo effects are controlled for. Meanwhile, for Western Europe, we can see that accounting for changes in tempo using the mean age at childbirth results in a more convex association between income and fertility.

**Table 2:** Country-by-year fixed effects, tempo-adjusted total fertility rate (TFR). *Source*: Eurostat, Statistical Offices, Cambridge Econometrics; own calculations

Independent variables	Europe (combined sample)	Western Europe	Eastern Europe
	(1)	(2)	(3)
Prior year compensation per capita (natural log)	− 1.120 (0.097)**	− 2.089 (0.262)**	− 1.754 (0.222)**
Prior year compensation per capita squared (natural log)	0.059 (0.005)**	0.109 (0.014)**	0.102 (0.014)**
Constant	7.131 (0.451)**	11.755 (1.235)**	8.875 (0.885)**
Inversion point (in 2005 Euros)	13,075 €	14,289 €	5324 €
Country-year interacted fixed effects	Yes	Yes	Yes
Number of observations	5205	4116	1089
Regions	256	200	56
Adjusted R-squared	0.769	0.762	0.764

Robust standard errors in parentheses

Employee compensation is defined as the total remuneration, in cash or in kind, payable by an employer to an employee in return for work done by the latter. It consists of wages and salaries, and of employers’ social contributions and is adjusted to 2005 Euros. Tempo-adjusted TFR is the adjusted total fertility rate, which is equal to TFR/(1 − ∆MACB), where ∆MACB is the annual change in the mean age at childbearing. Inversion points are calculated using coefficients rounded to the 8th decimal point

**p < 0.01, *p < 0.05, ^+^*p* < 0.1

### Robustness Checks

We implemented a number of robustness checks, which are discussed and presented in the online appendix. Here, we summarise briefly their results. Our robustness checks consisted of including regional fixed effects, using GDP per capita instead of employee compensation per capita, running separate models for the period before and after the onset of the 2007 recession, interacting income with regional indicators for regions in German-speaking and Southern European countries, and exploring whether spatial autocorrelation unaccounted for by our main models may bias the derived estimates (see also Belotti et al. 2016; Vitali and Billari 2017). The spatial models required a balanced panel, so that we reduced the observation period and excluded two countries with shorter time series (Romania and the Slovak Republic).

In our consistency checks for Western Europe, significance levels for our income variables were in some instances reduced. This includes, for example, the models with additional regional fixed effects and the GDP model with TFR as dependent variable. The results nevertheless remain generally robust across both fertility measures.^8^ When using GDP per capita, the estimated relationship was slightly less convex, but still statistically significant and with an estimated inversion point around 67,000 Euros per capita. The results for Eastern Europe are also quite robust. In contrast to Western Europe, however, we obtained in the tempo-adjusted models with additional regional fixed effects as well as in those models controlling for spatial autocorrelation reduced significance levels for our income covariates. For the tempo-adjusted model controlling for spatial autocorrelation, we even got non-statistically significant estimates for our income variables. However, in this model, the coefficient for the spatially clustered error term was also not significant. Thus, there is no indication to be concerned that the significant estimates for our income variables in our main model on the tempo-adjusted TFR without controls for spatial autocorrelation might be substantially biased due to unaccounted spatial autocorrelation. In our consistency checks with the balanced panel that excluded Romania, we show that the potential measurement bias in the Romanian data is not driving the outcomes of our main models which include Romania. But we remain cautious in interpreting the outcomes for Eastern Europe as support for the emergence of a convex relationship.

## Discussion and Conclusion

Recently proposed theoretical considerations (Esping-Andersen and Billari 2015; Goldscheider et al. 2015) and national-level empirical evidence (Myrskylä et al. 2009; Luci-Greulich and Thévenon 2014) suggest that at high levels of development the association between development and fertility might turn from negative to positive. However, the robustness and interpretation of these findings continue to be debated. In this paper, we sought to add the sub-national regional dimension to the discussion of a potential reversal in the relationship between income and fertility at high levels of development. The sub-national dimension helps to clarify the potential mechanisms through which development could be related to fertility, as it permits to control for national-level idiosyncrasies while also allowing for large variation in income.

The theoretical contribution of our paper is to point out a number of recent and current processes that could foster a weakening or a reversal of the long-standing negative relationship between economic development and fertility levels across sub-national regions in highly developed countries. These processes include in the area of family policies the replacement of more direct transfer payments with a new generation of policies that support the reconciliation of family and career goals; the growth in location-flexible working arrangements that decrease the opportunity costs of having children; and selective international and domestic migration patterns that may affect fertility directly in highly developed areas through the population composition and indirectly through effects on partner markets within outmigration areas. Our theoretical considerations for how these trends could shape associations between income and fertility are important for forming expectations on future regional fertility variation within highly developed countries.

Analysing data from 20 European countries between 1990 and 2012, we explored empirically for indications of an attenuation or turnaround of the long-standing negative relationship between fertility and economic development across sub-national regions. For most countries surveyed, we detected tendencies in this direction. However, the specific fertility and economic development levels at which tendencies towards a change in the relationship between fertility and economic development are observed vary substantially across countries. Our longitudinal regression analyses provided evidence for a convex association between income and fertility in Western and Eastern Europe. These findings seem particularly robust in Western Europe, which fits with our expectation that the emergence of such a relationship happens first in the most developed European countries. The estimated income level at which the association between income and fertility changes from negative to positive was much lower in the east than in the west, but varied in both regions depending on the model specification. Thus, similarly as Harttgen and Vollmer (2014), we cannot draw conclusions about any specific turning point. Furthermore, in many countries, the evidence is too weak to determine whether the association between income and fertility is transforming to strictly positive, or simply to one that is “not negative”, where income is less related to fertility differences across areas. We emphasise this qualification particularly for Eastern Europe, where only Poland and the Slovak Republic seem to have made the transition to a positive fertility–income relationship.

An important contributor to the fall and rise of fertility in European countries in the period we have analysed is fertility postponement, which first suppressed period fertility measures, before it boosted them as postponement slowed. It is disputed to what extent this pattern might explain the national-level inverse J-shaped association between fertility and development. Restrictions in data availability only allowed us to control for postponement by using information on mean age at childbirth, while we lacked data by parity. This may distort the results, as the changes in the timing of first births can differ from those observed for all births or for higher-order births. Prior research has suggested that the fertility rebound in Eastern Europe is largely driven by postponement (e.g. Goldstein et al. 2009). The outcomes of our admittedly crude attempt to control for postponement in Eastern Europe are in line with these findings as the convex fertility–income association gets weaker. However, both coefficients remain significant in the expected direction. In Western Europe, on the other hand, our attempt to control for postponement does not weaken the support for the existence of a convex relationship. We thus consider it unlikely that using a parity-specific control for postponement would significantly change the pattern identified for Western Europe. However, we remain nevertheless cautious with claims that postponement has no additional effect beyond what was identified above, as sub-national data are, for example, more affected by selective migration between regions and from abroad during the reproductive period of the life course. In addition, we also remain cautious about overemphasising the results for Eastern Europe. The observed “reversal” for Eastern European countries could be a function of unmeasured postponement effects in these areas, spatially uneven recovery from the post-communist recession, or issues in the measurements of regional fertility rates. We hope for future research to improve our understanding how aspects such as selective migration patterns might affect attempts to control for postponement at the sub-national regional level.

Our chosen empirical approach is well suited to study shifts in the fertility–income relationship at the sub-national regional level for a large number of European countries. However, data constraints did not allow us to explicitly test at a pan-European scale whether the observed shifts in the fertility–income association are indeed related to the processes outlined in our theoretical considerations. In order to study this in detail, we believe it is best to focus on countries for which register or register-like data are available. Such data allow for analyses with large samples of individual-level data at high geographic detail that include controls at the individual or contextual local level for aspects considered in our theoretical considerations (e.g. child care, flexible working arrangements, migration patterns, etc.). A recent example of such an approach is Wood et al. (2017). The patterns observed in our study are nevertheless consistent with our theoretical considerations. Among the Western European countries that currently have a positive or only slightly negative relationship, and that experienced the turnaround at relatively high fertility levels, are France, Belgium, Sweden, and Norway. These countries were among the forerunners in the introduction of family policies aimed at supporting parents in reconciling their family and career goals (Gauthier 2002; PERFAR 2017). Another pattern consistent with our considerations is that in Austria a positive relationship existed throughout the period, but at very low fertility levels. Existing research has attributed this distinctive pattern to selective international migration to the most developed region of Vienna (Zeman et al. 2010).

One additional restriction is that we only have access to income data which was measured at the place of work, while a measurement at the place of residence would be preferable. This might create biases when big cities or metropolitan areas constitute their own regions into which people commute into for work. However, this data limitation would bias against the identification of a convex relationship shown in Tables 1 and 2, as high incomes generated in the most developed regions would be used for realising fertility intentions in less developed neighbouring regions. For future research, it might also be promising to perform a similar study on male fertility, as many less developed peripheral areas have male-dominated sex ratios among the inhabitants of childbearing age. Thus, the observed tendencies towards an attenuation or reversal of the association might even be stronger if male fertility was considered.

Overall, our paper provides theoretical support for the view that a turnaround in the relationship between fertility and economic development might occur as well at the sub-national level in highly developed countries. Our empirical analysis indicates that such tendencies are already visible, particularly in Western Europe. However, it is too early to draw definite conclusions whether we are indeed witnessing a turnaround to a genuine positive relationship between income and fertility, or simply the emergence of a less or non-negative relationship. As sub-national parity-specific register or register-like data become increasingly available across Europe, future research might be able to investigate shifts in the sub-national fertility–income association with more detailed data in the years to come.

### Electronic supplementary material

Below is the link to the electronic supplementary material.

Supplementary material 1 (DOCX 121 kb)

## Data Appendix

In selecting our sample of sub-national regions, we relied on the NUTS classification system of the European Union that distinguishes countries and sub-national regions of comparable population size across Europe. NUTS 1 generally represents regional divisions with populations between three and seven million persons, NUTS 2 regional divisions with populations between about 800,000 and three million persons, and NUTS 3 areas with populations between about 150,000 and 800,000 persons. We utilise NUTS 2 level data, as this is the lowest level of division for which the required fertility and economic data are available. Two limitations of the NUTS classification are that the regions do not necessarily reflect long-standing administrative divisions within countries, and that the population sizes of regions are not always within the proposed ranges. But it is a widely used and generally consistent geographic classification system.

In our analyses on the association between income and fertility, we require the two variables of per capita income and per capita income squared. Since we use a fixed effects approach, for any given country at least three different regions are needed for an estimate. To ensure at least one degree of freedom, we therefore exclude the following EU/EFTA countries with less than four regions from the analysis: Croatia, Cyprus, Estonia, Iceland, Ireland, Latvia, Liechtenstein, Lithuania, Luxembourg, Malta, and Slovenia. In addition, Switzerland is excluded due to a lack of income data. The analysed Western European countries/areas are Austria, Belgium, Denmark, Finland, France, former West Germany, Greece, Italy, the Netherlands, Norway, Portugal, Spain, Sweden, and the UK. In Eastern Europe, we cover Bulgaria, the Czech Republic, former East Germany, Hungary, Poland, Romania, and the Slovak Republic.

In addition to the above countries, we also exclude a handful of unusual NUTS 2 regions. We omit these regions because they are either geographically isolated and/or culturally deviating from the countries’ mainland. These are the Swedish-speaking Åland Islands in Finland, the Canary Islands and the North African exclaves of Ceuta and Melilla in Spain, and the Azores and Madeira in Portugal. For Germany, we exclude the region of Berlin from the panel data models, as the data do not allow distinguishing former East from West Berlin.

The demographic data on the births by five-year age groups and the female populations by five-year age groups have mainly been obtained from the Eurostat web database (final datasets were last updated as of April 14, 2015 (population data) and April 29, 2015 (birth data)). In addition, we used data from national statistical offices to fill large time series gaps for a number of countries during the period of observation. Data from the national statistical offices also allowed us to correct inconsistencies in the Eurostat data. For example, we noticed an anomaly in the Finnish birth data available from Eurostat, which in 1996 use a maternal age seemingly derived differently than the rest of the series. This anomaly disappeared, when we replaced the Eurostat data with data available in the StatFin database of Statistics Finland. Table 3 contains information on temporal coverage and on the alternative sources from which we obtained data to complement and correct data that are available through Eurostat. It also provides details on slight inconsistencies in the data for a small number of regions.

**Table 3:** Demographic data sources

Country	Years	Notes
Austria	1990–2012	Censuses: 1991 (C), 2001 (C), 2011 (RB)
Belgium	1990–2012	Censuses: 1991 (C), 2001 (C), 2011 (RB)
Bulgaria	1990–2012	Censuses: 1992 (C), 2001 (C), 2011 (C)
Czech Republic	1992–2012	Censuses: 1991 (C), 2001 (C), 2011 (C)
Denmark	1990–2012	For the Danish NUTS 2 regions newly formed in 2007, Eurostat provides only population data dating back to 1990, and no birth data. The latter have been derived based on municipality-level data from the StatBank of Statistics Denmark Censuses: Annual data derived from register
France	1990–2012	Censuses: 1990 (C), 1999 (C), 2004–2008 (RC), 2009–2014 (RC)
Finland	1990–2012	In the Eurostat data for the births in 1996 the age of the mother was derived based on a different standard than for the rest of the series. This was confirmed by a comparison of the data with data available in the StatFin database of Statistics Finland. Thus, we replaced the 1996 birth data with data directly obtained from the StatFin database Censuses: Annual data derived from register
Germany (West/East)	1991–2012	For the period 1991–1999, the data were obtained from individual-level counts derived from the birth register (FDZ 2015). Birth register data are missing for the regions of Mecklenburg-Vorpommern between 1991 and 1994 and Saarland in 1991. In these cases we obtained data from published statistics in which the age of the mother had been derived by subtracting the birth year of the mother from the birth year of the child Economic data from Cambridge Econometrics for the regions Chemnitz and Leipzig are based on an old regional classification where the district of Döbeln is included in Leipzig, not Chemnitz. As such we derived the demographic data based on this old set-up. However, for the years 2011 and 2012 we only were able to obtain fertility data for the new set-up. But this should not have an effect on our estimates as the regions Chemnitz and Leipzig had in this period very similar fertility trends and levels Censuses: 1981 (East, C), 1987 (West, C), 2011 (RB)
Greece	1990–2012	Censuses: 1991 (C), 2001 (C), 2011 (C)
Hungary	1990–2012	Censuses: 1990 (C), 2001 (C), 2011 (C)
Italy	1990–2012	Emilia-Romagna and Marche exchanged seven municipalities in 2009 and as such Eurostat provides no time series before 2000. We were not able to reconstruct the data for the 1990s based on the current regional boundaries, but since the administrative reform affected only small parts of the population (less than 0.5% of that of Emilia-Romagna and 1.2% of that of Marche), having a small change in the regional set-up of these two regions between 1999 and 2000 should not significantly affect any estimates. For the 1990s, we obtained the data for these two regions from a database by ISTAT, which provides regional fertility rates by single ages For Lazio, the age-specific fertility rates derived from Eurostat for 1998 deviated strongly from the time trend and the initially published Italian statistics. Data for Lazio for 1998 were replaced by data from ISTAT Censuses: 1991 (C), 2001 (C), 2011 (RB)
The Netherlands	1990–2012	For the period 1990–2000 Eurostat does not provide regional data for the Netherlands. These data were derived directly from Statistics Netherlands. For these data the age of the mother is derived differently than in the Eurostat data-probably by subtracting the birth year of the mother from the birth year of the child Censuses: 1991 (RB), 2001 (RB), 2011 (RB)
Norway	1990–2012	1990 (C, in part RB), 2001 (C, in part RB), 2011 (RB)
Poland	1991–2012	Censuses: 1988 (C), 2002 (C), 2011 (C)
Portugal	1992–2012	Censuses: 1991 (C), 2001 (C), 2011 (C)
Romania	1993–2012	For the births in 1998, 2000, and 2010 the age of the mother seems to have been derived based on a different standard than for the rest of the series. This was confirmed by a comparison with the time series data available in the TEMPO Online database of Statistics Romania. Thus, we replaced the birth data for these 3 years with the data directly obtained from Statistics Romania. Eurostat only has regional data from 1997 onward, while the TEMPO Online database of Statistics Romania contains a series from 1993 onward which we included in the analysis Censuses: 1992 (C), 2002 (C), 2011 (C)
Slovak Republic	1996–2012	Censuses: 1991 (C), 2001 (C), 2011 (C)
Spain	1990–2012	Censuses: 1991 (C), 2001 (C), 2011 (RB)
Sweden	1990–2012	Censuses: Annual data derived from register, 1990 (C)
UK	1991–2012	Eurostat does not provide regional UK data prior to 2000. Data for the 1990s were derived from local population statistics obtained from the UK data archive. Economic data from Cambridge Econometrics for the regions Cheshire and Merseyside are based on an old regional classification where the city of Halton is included in Cheshire, not Merseyside. As such the demographic data are derived based on this set-up For Scotland there is a small break in the regional definition in 2000, and it was not possible to reconstruct data for the previous period based on the current regional division. The changes were a movement of the islands of North Ayrshire from the region Islands and Highlands to Southwest Scotland and a movement of Helensburgh and Lomond from Southwest Scotland to the region Islands and Highlands. For the period before 2000, both birth counts and population data reflect the old regional division Among the two NUTS 2 regions in Wales, up to 1994 the births for a number of small hamlets and settlements are assigned to the wrong NUTS 2 region, while the population at risk is assigned correctly. This should just have minor effects as these hamlets are very small relative to the total population of the two NUTS 2 regions of Wales Censuses: 1991 (C), 2001 (C), 2011 (C)

*C* Census, *RB* register-based census, *RC* rolling census, in part register-based

While the registration of births is quite reliable across European countries, the quality of population data used to calculate the fertility rates might vary across our dataset of 256 regions. During our observation period, most countries were still relying on a classical approach to derive population statistics with censuses and intercensal population estimates based on registered vital and migration events. A major source of error in the production of intercensal population estimates is the under-registration of migration events, which can lead to bias in the population data that might be particularly high at the end of the intercensal period. The registration of migration events is particularly problematic when migrants move abroad (Raymer et al. 2011). If they do not register their departure, it can take years to detect their absence. During our observation period, the Eastern European countries in particular experienced substantial outmigration. High rates occurred immediately after the fall of the Iron Curtain in the early 1990s, and after the 2004 and 2007 EU enlargement rounds (Eurostat). In addition, Southern European countries faced non-negligible outmigration after the onset of the 2007 recession, which was likely in part not registered.

Fortunately, population data for most of these countries have been retrospectively adjusted by the statistical offices based on the outcomes of the 2001 and 2011 census rounds. For the last census round this was true for Bulgaria, the Czech Republic, Greece, Italy, Portugal, the Slovak Republic, Spain, and the UK. Other countries implemented backward adjustments for some years (e.g. Austria for 2008–2011; and Poland for 2010–2011). Among the Eastern and Southern European countries, only Hungary, Poland for the period 2001–2009, and Romania lack backward-adjusted population numbers. Another potentially problematic country is Germany that had a long intercensal period between the East German census of 1981 and the West German census of 1987, and the first census after reunification in 2011. Due to the long intercensal period, the German Federal Statistical Office decided not to provide backward adjustments by age for the intercensal period. In Germany, the regional adjustments implemented after the census suggest that both in eastern and western Germany the population over-count was highest in high developed areas, implying that for these areas the fertility rates are downward biased. This would then make it more difficult to identify a convex relationship. But the deviations at the end of the long intercensal period are generally small with a mean of − 1.6% deviation among the main childbearing age groups between 20 and 45 with minimum values of − 5% and only very few positive values. This is in line with outcomes of an intercensal adjustment of the population data for eastern and western Germany implemented as part of the Human Fertility Database project. Klüsener et al. (2018) concluded that German fertility trends were only marginally affected (the highest deviation between the original and adjusted TFR was 0.03 in eastern Germany if all years of the intercensal period are compared). If the deviation pattern for Hungary is similar to the Czech Republic and the Slovak Republic, where higher over-counts of up to 5% were registered in the most developed areas, the bias in Hungary would also rather work against identifying a convex relationship. For Poland, the capital region was both the most developed area and had the lowest over-count, so the bias might increase the likeliness to identify a convex relationship. However, only one region (Opole) exhibited a strong population over-count, while the others deviated only by a maximum of three per cent points from the deviation registered in Warsaw.

More serious data quality issues are evident in the Romanian data, for which we have only backward-adjusted numbers for the national level available for the intercensal period between 2002 and 2011. The Romanian regional data show at the end of the intercensal period deviations of up to 20% among females aged 20–34 years. The breaks in the regional Romanian population time trends due to the corrections implemented after the 2011 census suggest that population numbers were too high in low developed outmigration areas and too low in the high developed capital area. This fits with the regional pattern in neighbouring Bulgaria, where we are able to contrast the originally published with the backward-corrected population estimates. Romania is the only obvious example where we witness a strong bias in our data towards the likeliness of identifying a convex relationship. However, even in countries whose data have been backward-adjusted, there may be inconsistencies and potential data quality issues. This was the case for the 2011 census in Poland, for instance (Tymicki and Zeman 2017). As such, the Eastern European findings, as well as those for Southern Europe after the onset of the 2007 crisis, should be interpreted with some caution.

The TFR is calculated by aggregating the female age-specific fertility rates (births per 1000 women of a specific age) across the six five-year age groups between the fertile ages of 15 and 44. It can be interpreted as the average number of children a woman would have if the age-specific fertility rates of a given year would stay constant over the whole reproductive life-span of that woman. To create a tempo-adjusted TFR, the approach outlined in Bongaarts and Sobotka (2012) represents arguably the most sophisticated approach suggested in the literature, but requires parity-specific fertility by age for the different regions. As these data are not available, we use a second-best method of weighting by the average age at childbearing. This method has been described and evaluated by Potančoková et al. (2008). Specifically, we estimate the tempo-adjusted TFR as suggested by Bongaarts and Feeney (1998), by calculating $\widehat{TFR}^{*}$ = TFR/(1-∆MACB), where ∆MACB is the annual change in the mean age at childbearing.

Economic data were collected by Cambridge Econometrics (CE) primarily from Eurostat, and then adjusted to 2005 Euros using purchasing power parity deflators. A discussion why we decided to use employee compensation per capita is presented in the main text. For documentation of the economic data from CE, we refer interested readers to their data write-up available online (Cambridge Econometrics 2015).

Table 4 provides summary statistics for the set of European countries used in our analysis. Column (1) of Table 4 shows the total number of sub-national NUTS 2 regions by country, and the total number of regions included in our models. Column (2) provides information on the periods for which fertility data for the given year and economic data for the preceding year are available. Columns (3), (4), (5), and (6) of Table 4 list the average per capita GDP, the average employee compensation per capita, the average TFR, and the average tempo-adjusted TFR. We emphasise that these averages are not the averages of the different countries, but the averages *across all their regions over the whole time period*. Per capita GDP is defined as Total Gross Value Added, plus taxes, less subsidies on products, divided by the region’s population. Employee compensation, which is our preferred measure of per capita income as it accounts for household income levels better than GDP, is defined as the total remuneration, in cash or in kind, payable by an employer to an employee in return for work done by the latter. It consists of wages and salaries, and of employers’ social contributions. Both GDP and employee compensation are adjusted to 2005 Euros. We present the GDP and employee compensation averages side by side to show how the former compares to the latter. In general, GDP per capita is about twice that of employee compensation, but the exact ratio varies slightly across countries. Comparison of the average TFR and average tempo-adjusted TFR gives a sense of which countries experienced significant degrees of postponement. Postponement has been a factor across all of the different countries in our sample, but its magnitude seems to vary across areas. In Belgium, for instance, the two averages are not that far apart (1.71 to 1.85). On the other hand, the average of the regional tempo-adjusted fertility rates in the Czech Republic is nearly 30% higher than the average of the regional period fertility rates indicating that postponement has played a much more important role there.

**Table 4:** Country list and summary statistics. *Source*: Eurostat, Statistical Offices, Cambridge Econometrics; own calculations

Country	NUTS 2 regions all/used	Years	Avg. GDP per capita (2005 €)	Avg. emp. comp. per capita (2005 €)	Avg. total fertility rate (TFR)	Avg. tempo-adjusted TFR
	(1)	(2)	(3)	(4)	(5)	(6)
Austria	9/9	1990–2012	24,487	12,457	1.42	1.66
Belgium	11/11	1990–2012	23,564	10,627	1.71	1.85
Bulgaria	6/6	1991–2012	2622	1029	1.44	1.66
Czech Republic	8/8	1992–2012	9079	3728	1.35	1.74
Denmark	5/5	1990–2012	31,206	16,733	1.81	2.02
Finland	5/4	1990–2012	22,001	11,086	1.86	1.99
France	22/22	1990–2012	21,398	12,806	1.81	1.97
Germany, East	8/7	1992–2012	17,751	9791	1.19	1.50
Germany, West	30/30	1991–2012	26,249	13,980	1.38	1.57
Greece	13/13	1990–2012	13,448	4178	1.39	1.67
Hungary	7/7	1991–2012	6676	3088	1.43	1.68
Italy	21/21	1990–2012	21,262	9000	1.29	1.44
The Netherlands	12/12	1990–2012	25,764	12,763	1.71	1.79
Norway	7/7	1990–2012	40,193	18,588	1.87	2.07
Poland	16/16	1991–2012	5192	2012	1.47	1.62
Portugal	7/5	1992–2012	11,992	5641	1.43	1.65
Romania	8/8	1993–2012	3358	1327	1.33	1.51
Slovak Republic	4/4	1996–2012	7431	2907	1.32	1.62
Spain	19/16	1990–2012	17,025	7398	1.25	1.46
Sweden	8/8	1990–2012	26,262	14,552	1.81	1.96
UK	37/37	1991–2012	23,414	12,567	1.77	1.95

Average values (Avg.) in the columns (3)–(6) of the table represent averages across all regions and all years for the different countries presented. Emp. comp. is employee compensation, defined as the total remuneration, in cash or in kind, payable by an employer to an employee in return for work done by the latter. It consists of wages and salaries, and of employers’ social contributions. GDP is the Gross Domestic Product, defined as Total Gross Value Added plus taxes less subsidies on products. Both employee compensation and GDP are adjusted to 2005 Euros. TFR is the period total fertility rate. The tempo-adjusted TFR is equal to TFR/(1 − ∆MACB), where ∆MACB is the annual change in the mean age at childbearing. German, Finnish, Portuguese, and Spanish regions are excluded because they are non-mainland regions and/or are different culturally and politically. For Finland, these regions include the Åland islands; for Germany, Berlin; for Portugal, the Acores and Madeira, which constitute island regions in the Atlantic; and for Spain, the North African exclaves of Ceuta and Melilla and the Canary Islands in the Atlantic

## Footnotes

1 These countries are Austria, Belgium, Bulgaria, the Czech Republic, Denmark, Finland, France, Germany, Greece, Hungary, Italy, the Netherlands, Norway, Poland, Portugal, Romania, the Slovak Republic, Spain, Sweden, and the United Kingdom (UK).

2 Capital regions and centres of economic activity are commonly characterised by high degrees of urbanisation. The only significant exception where highly urbanised areas might not be among the most developed areas of a country is among regions that industrialised early and relied heavily on economic sectors that have lost relevance over the last 50 years (e.g. coal mining and the steel industry). Examples of such regions are the German Ruhr area, northeastern England, central Belgium, and Polish Upper Silesia (see also Hospers 2004).

3 Results from the analyses below were not sensitive to adjusting income per capita to income per worker or income per active population.

4 The division in Western and Eastern European countries is analogous to the one applied for Figs. 1 and 2.

5 Specifically, we estimate the Bongaarts and Feeney (1998) tempo-adjusted TFR by calculating $\widehat{TFR}^{*}$ = TFR/(1 − ∆MACB), where ∆MACB is the annual change in the mean age at childbearing.

6 According to Potančoková et al. (2008), “relatively low” fertility is a TFR below 2.0.

7 The mean age at childbearing is smoothed using a three-year moving average of *t − 1*, *t*, and *t + 1*.

8 One exceptional outcome was the prediction of a concave relationship in the models for Western Europe in the period after the onset of the 2007 recession. However, although of different signs, plotting the trajectories implied by the coefficients on income when using tempo-adjusted fertility yields relatively similar trends between the two contrasted periods over the observed range of incomes per capita. All that said, the models for the recession years are likely underpowered with insufficient sample sizes, and more importantly, the results using the pre-2007 period are robust.

## References

[CR1] Abramitzky R, Boustan LP, Eriksson K (2012). Europe’s tired, poor, huddled masses: Self-selection and economic outcomes in the age of mass migration. American Economic Review.

[CR2] Alam A, Murthi M, Yemtsov R, Murrugarra E, Dudwick N, Hamilton E, Tiongson E (2005). Growth, poverty, and inequality: Eastern Europe and the former Soviet Union.

[CR3] Baizan P (2009). Regional child care availability and fertility decisions in Spain. Demographic Research.

[CR4] Basten S, Huinink J, Klüsener S (2011). Spatial variation of sub-national fertility trends in Austria, Germany and Switzerland. Comparative Population Studies.

[CR5] Becker GS, National Bureau of Economic Research (1960). An economic analysis of fertility. Demographic and economic changes in developed countries.

[CR6] Begall K, Mills M (2011). The impact of subjective work control, job strain and work–family conflict on fertility intentions: A European comparison. European Journal of Population.

[CR7] Belotti, F., Hughes, G., & Mortari, A. P. (2016). *Spatial panel data models using Stata*. Rome: University of Rome “Tor Vergata”. CEIS Tor Vergata Research Paper Series (Vol. 14, Issue 5, No. 373).

[CR8] Beninger, A., & Carter, N. M. (2013). *The great debate: Flexibility vs. face time—Busting the myths behind flexible work arrangements*. Catalyst. http://www.catalyst.org/system/files/the_great_debate_flexibility_vs_face_time.pdf. Accessed February 1, 2018.

[CR9] Billari FC, Dalla-Zuanna G (2011). Is replacement migration actually taking place in low fertility countries?. Genus.

[CR10] Blau FD, Mackie C (2017). The economic and fiscal consequences of immigration.

[CR11] Blythell D, O’Brien P, Quinault R (1993). Women in the workforce. The industrial revolution and British society.

[CR12] Bongaarts J, Feeney G (1998). On the quantum and tempo of fertility. Population and Development Review.

[CR13] Bongaarts J, Sobotka T (2012). A demographic explanation for the recent rise in European fertility. Population and Development Review.

[CR14] Bonifazi C, Okólski M, Schoorl J, Simon P (2008). International migration in Europe: New trends and new methods of analysis.

[CR15] Burger O, DeLong JP (2016). What if fertility decline is not permanent? The need for an evolutionarily informed approach to understanding low fertility. Philosophical Transactions of the Royal Society B: Biological Sciences.

[CR20] Cambridge Econometrics (2015). European Regional Database.

[CR16] Cleland J, Wilson C (1987). Demand theories of the fertility transition: An iconoclastic view. Population Studies.

[CR17] de Meester E, van Ham M (2009). Symmetry and asymmetry in working and commuting arrangements between partners in the Netherlands: Does the residential context matter?. Environment and Planning A.

[CR18] Decroly JM, Grasland C (1993). Boundaries, political systems and fertility in Europe. Population: An English Selection.

[CR19] Dribe M, Scalone F (2014). Social class and net fertility before, during, and after the demographic transition: A micro-level analysis of Sweden 1880–1970. Demographic Research.

[CR21] Esping-Andersen G, Billari FC (2015). Re-theorizing family demographics. Population and Development Review.

[CR22] FDZ [Forschungsdatenzentrum] der Statistischen Ämter des Bundes und der Länder (2015). Geburtenstatistik 1991–2010.

[CR23] Gauthier AH (2002). Family policies in industrialized countries: Is there convergence?. Population-E.

[CR24] Gauthier AH (2007). The impact of family policies on fertility in industrialized countries: A review of the literature. Population Research and Policy Review.

[CR25] Goldscheider F, Bernhardt E, Lappegård T (2015). The gender revolution: A framework for understanding changing family and demographic behavior. Population and Development Review.

[CR26] Goldstein JR, Klüsener S (2014). Spatial analysis of the causes of fertility decline in Prussia. Population and Development Review.

[CR27] Goldstein JR, Kreyenfeld M (2011). Has East Germany overtaken West Germany? Recent trends in order-specific fertility. Population and Development Review.

[CR28] Goldstein JR, Sobotka T, Jasilioniene A (2009). The end of “lowest-low” fertility?. Population and Development Review.

[CR29] Guinnane TW (2011). The historical fertility transition: A guide for economists. Journal of Economic Literature.

[CR30] Harttgen K, Vollmer S (2014). A reversal in the relationship of human development with fertility?. Demography.

[CR31] Hayford AM (1974). The geography of women: An historical introduction. Antipode.

[CR32] Hospers G-J (2004). Restructuring Europe’s rustbelt: The case of the German Ruhrgebiet. Intereconomics.

[CR33] Kalwij A (2010). The impact of family policy expenditure on fertility in western Europe. Demography.

[CR34] Kirk D (1996). Demographic transition theory. Population Studies.

[CR35] Klüsener, S., Grigoriev, P., Scholz, R. D., & Jdanov, D. A. (2018). Adjusting inter-censal population estimates for Germany 1987–2011: Approaches and impact on demographic indicators. *Comparative Population Studies* (accepted for publication).

[CR36] Klüsener S, Neels K, Kreyenfeld M (2013). Family policies and the Western European fertility divide: Insights from a natural experiment in Belgium. Population and Development Review.

[CR37] Klüsener, S., Scalone, F., & Dribe, M. (2014). Spatial vs. social distance in the diffusion of fertility decline: Evidence from Sweden 1880–1900. In *Paper presented at the Annual Meeting of the Population Association of America 2014 in Boston*, MA, 1–3 May 2014.

[CR38] Kocka J (1990). Arbeitsverhältnisse und Arbeiterexistenzen: Grundlagen der Klassenbildung im 19. Jahrhundert.

[CR39] Krugman P (1998). What’s new about the new economic geography?. Oxford Review of Economic Policy.

[CR40] Lawson DW, Mace R (2011). Parental investment and the optimization of human family size. Philosophical Transactions of the Royal Society B: Biological Sciences.

[CR41] Lesthaeghe R (2010). The unfolding story of the second demographic transition. Population and Development Review.

[CR42] Lipset SM, Bendix R (1991). Social mobility in industrial society.

[CR43] Lorimer F (1945). Issues of population policy. The Annals of the American Academy of Political and Social Science.

[CR44] Luci-Greulich A, Thévenon O (2014). Does economic advancement ‘cause’ a re-increase in fertility? An empirical analysis for OECD countries (1960–2007). European Journal of Population.

[CR45] Macours K, Swinnen JFM (2008). Rural-urban poverty differences in transition countries. World Development.

[CR46] Mehta NK, Elo IT (2012). Migrant selection and the health of US immigrants from the former Soviet Union. Demography.

[CR47] Myrskylä M, Goldstein JR (2013). Probabilistic forecasting using stochastic diffusion models, with applications to cohort processes of marriage and fertility. Demography.

[CR48] Myrskylä M, Goldstein JR, Cheng Y-HA (2013). New cohort fertility forecasts for the developed world: Rises, falls and reversals. Population and Development Review.

[CR49] Myrskylä M, Kohler H-P, Billari FC (2009). Advances in development reverse fertility declines. Nature.

[CR50] Myrskylä, M., Kohler, H.-P., & Billari, F. C. (2011). *High development and fertility: Fertility at older reproductive ages and gender equality explain the positive link*. Rostock: Max Planck Institute for Demographic Research. MPIDR Working Paper WP 2011-017, revised November 2013.

[CR51] Neyer G, Andersson G (2008). Consequences of family policies on childbearing behavior: Effects or artifacts?. Population and Development Review.

[CR52] Oppenheim Mason K (1997). Explaining fertility transitions. Demography.

[CR53] PERFAR. (2017). *Population Europe Resource Finder & Archive*. Berlin: Population Europe. http://www.perfar.eu/. Accessed April 1, 2017.

[CR54] Potančoková, M., Sobotka, T., & Philipov, D. (2008). Estimating tempo effect and adjusted TFR: Documentation to the European Demographic Datasheet 2008 (July 2008). Vienna: Vienna Institute of Demography. https://www.oeaw.ac.at/fileadmin/subsites/Institute/VID/PDF/Publications/Datasheet/DS2008/Tempo_effect_Documentation_VID_23-07-2008.pdf. Accessed February 1, 2018.

[CR55] Raymer J, de Beer J, van der Erf R (2011). Putting the pieces of the puzzle together: Age and sex-specific estimates of migration amongst countries in the EU/EFTA, 2002–2007. European Journal of Population.

[CR56] Ryabov I (2015). On the relationship between development and fertility: The case of the United States. Comparative Population Studies.

[CR57] Schmertmann C, Zagheni E, Goldstein JR, Myrskylä M (2014). Bayesian forecasting of cohort fertility. Journal of the American Statistical Association.

[CR58] Skirbekk V (2008). Fertility trends by social status. Demographic Research.

[CR59] Sobotka T (2003). Re-emerging diversity: Rapid fertility changes in Central and Eastern Europe after the collapse of the communist regimes. Population-E.

[CR60] Thévenon O (2011). Family policies in OECD countries: A comparative analysis. Population and Development Review.

[CR61] Tymicki, K., & Zeman, K. (2017). *Human Fertility Database documentation: Poland* (Last revision: 4 October 2017). http://www.humanfertility.org/Docs/POL/POLcom.pdf. Accessed February 1, 2018.

[CR62] Vitali A, Billari FC (2017). Changing determinants of low fertility and diffusion: A spatial analysis for Italy. Population, Space and Place.

[CR63] Watkins SC (1991). From provinces into nations: Demographic integration in Western Europe, 1870–1960.

[CR64] Wigglesworth, R. (2016). *Pensions and ageing populations: The problem explained*. The Financial Times, 26 August 2016.

[CR65] Wilson, B. (2013). Disentangling the quantum and tempo of immigrant fertility. In *Paper presented at the 7th International Conference on Population Geographies in Groningen, The Netherlands*, 25–28 June 2013.

[CR66] Wood, J., Klüsener, S., Neels, K., & Myrskylä, M. (2017). *Is a positive link between human development and fertility attainable? Insights from the Belgian vanguard case*. Rostock: Max Planck Institute for Demographic Research. MPIDR Working Paper WP 2017-014—revised January 2018.

[CR67] Zeihan P (2014). The accidental superpower: The next generation of American preeminence and the coming global disorder.

[CR68] Zeman K, Sobotka T, Gisser R, Winkler-Dworak M, Lutz W (2010). Geburtenbarometer Vienna: An overview report.

