# Supplementary material for: Is a Positive Relationship Between Fertility and Economic Development Emerging at the Sub-National Regional Level? Theoretical Considerations and Evidence from Europe
Source: Eur J Popul. 2018 May 8;35(3):487–518. doi: 10.1007/s10680-018-9485-1 (PMC6639514; doi:10.1007/s10680-018-9485-1)
Supplement: Supplementary file 1 — Supplementary material 1 (DOCX 121 kb) [file 10680_2018_9485_MOESM1_ESM.docx]

**Is a Positive Relationship between Fertility and Economic Development Emerging at the Sub-National Regional Level?**

**Theoretical Considerations and Evidence from Europe**

Jonathan Fox, Sebastian Klüsener, Mikko Myrskylä

**Online Appendix: Robustness Checks**

This online appendix presents a series of consistency checks for the model estimates in Tables 1 and 2. These alternative specifications include (i) adding regional fixed effects to the model, (ii) replacing employee compensation per capita with Gross Domestic Product (GDP) per capita, (iii) estimating the model separately for the years prior to and following the onset of the 2007 financial crisis, (iv) controlling for membership in a German-speaking or Southern European country, and (v) controlling for the potential bias introduced by spatial autocorrelation that might remain unaccounted for in our main models.

**Regional fixed effects model**

If regions within countries exhibit heterogeneity in factors related to both income and fertility, then estimates from the models presented in the main text might be biased and inconsistent. To control for this unobserved heterogeneity across sub-national regions that is constant through time, this section presents sensitivity checks based on the model in equation (1). To this model, we include regional fixed effects in addition to the country-by-year fixed effects. The estimating equation is defined as:

|  | $y_{i,c,t}=\chi_{c}*\lambda_{t}+\lambda_{i}+\beta_{1}\ln\left( {inc}_{i.c, t-1} \right)+\beta_{2}{\ln\left( {inc}_{i,c,t-1} \right)}^{2}+\varepsilon_{i,c,t}$ | (OA1) |
| --- | --- | --- |

where $y_{i,c,t}$ is the outcome of interest (either total fertility rate (TFR) or the tempo-adjusted TFR), and $\lambda_{i}$ is the regional fixed effects term. We present estimates from this model in Table OA1.

With the inclusion of the regional fixed effects, the results are qualitatively the same for Western and Eastern Europe. Some minor differences nevertheless exist between the models shown in Table OA1 and the results from the primary models presented in Tables 1 and 2. Firstly, the precision of the coefficient estimates on income and income squared is attenuated. This results in a reduction of the statistical significance of the estimated income variable coefficients. However, all remain statistically significant at the 10 percent level. Secondly, the coefficient on income squared for the combined European sample using tempo-adjusted fertility is attenuated (resulting in less convexity) and, although nearly so, is not statistically significant at the 10 percent level. We nevertheless caution against conclusions based on the combined European sample, given the differences between Western and Eastern Europe as discussed in the main text. Lastly, the inversion points in the regional fixed effects models are estimated at a lower income level in the models using TFR, and the ones for Eastern Europe using tempo-adjusted TFR. The inversion point occurs at a greater income level for Western Europe in the tempo-adjusted model. The fluctuation of the estimated inversion point demonstrates the uncertainty surrounding the estimation of specific turnaround points.

A comparison of the results in Table OA1 to those in Tables 1 and 2 highlights the significant differences between Western and Eastern Europe. Once the unobserved regional fixed effects are controlled for, the estimated relationships between income and fertility in Western and Eastern Europe become more similar. If we contrast the results from the TFR models to the tempo-adjusted TFR models, they indicate unobserved regional differences related to postponement. After controlling for the regional fixed effects, the estimated relationships between income and fertility and income and tempo-adjusted fertility also become more similar.

Although heterogeneity exists among regions within countries, and some of that heterogeneity seems related to both fertility and income, the coefficients presented in Tables 1 and 2 are all within the confidence intervals of their counterpart estimates presented in Table OA1. Their sizes and the associated inversion points are somewhat affected, but qualitatively the conclusions remain as discussed in the main text.

**Using GDP instead of employee compensation as the measure for income**

In this sensitivity check, we apply the model in equation (1), but replace employment compensation per capita with GDP per capita as the measure of income. Regional GDP is calculated as Total Gross Value Added, plus taxes and less subsidies, and captures total income generated by both firms and households. Employee compensation per capita, on the other hand, consists of wages and salaries, and of employers' social contributions. We present the outcomes of this sensitivity check in Table OA2.

In comparison to the results of our main models in Tables 1 and 2, there exist slight differences in the coefficient estimates, and the estimated reversal point is increased. For example, when we use the TFR as dependent variable, the statistical significance is attenuated for the Western European sample, but remains significant at the 5 percent level. None of these differences are statistically significant, however.

**Contrasting the sub-periods before and after the onset of the 2007 financial crisis and subsequent global recession**

In another sensitivity check we estimate the model presented in equation (1) separately for the periods 1990–2007 and 2008–2012 (with income at a one-year lag from 1989–2006 and 2007–2011). The main motivation for this consistency check is to explore whether estimates for the relationship between income and fertility vary between the periods before and after the onset of the 2007 economic recession. By including the crisis years into the sample without explicitly controlling for their effect, we had parameterised that effect into being symmetric. If, for example, declines in income per capita affect fertility rates differently than increases in income per capita, then the recession years might have a bearing on our presented results. To explore this, we estimate the models separately for the two periods, with focus on the pre-crisis period to verify that the results presented in the main text were not driven by the recent financial crisis and subsequent global recession.

Tables OA3A and OA3B present these results using the TFR and the tempo-adjusted TFR, respectively. They indicate that the estimates given in Tables 1 and 2 are robust to the removal of the recessionary period, as they are statistically indistinguishable and similar in magnitude to their counterparts in Tables OA3A and OA3B. The results for Western Europe become slightly more convex after omitting the economic recession years, an understandable effect when looking at the coefficient estimates for Western European countries for the years 2008–2012. The relationship between income and fertility during this period is estimated as *concave* for the Western European countries when the TFR is the dependent variable (Table OA3A), indicating that income and fertility were positively linked for lower incomes but negatively linked for higher incomes. From the results using the tempo-adjusted TFR (Table OA3B), however, the concave relationship present for Western Europe using TFR is no longer statistically significant. In addition, although the coefficients on income are of different signs, plotting their implied trajectories when using tempo-adjusted fertility yields similar trends between the two contrasted periods over the observed range of incomes per capita. That said, the models for the recession years are likely underpowered with insufficient sample sizes, so we caution against placing too much stock in the results without further investigation. As such, while we find the result for Western Europe during the crisis period interesting, especially when compared to the Eastern European countries which seem to exhibit a different pattern of fertility in response to the crisis, a full analysis of the reasons behind these results is beyond the scope of this paper and awaits future research. As mentioned above, the most important conclusion we draw from this consistency check is that the results are robust to the exclusion of the crises years.

**Controlling for membership in German-speaking and Southern European countries**

For Western Europe, subgroups of countries tend to cluster in their variation in fertility patterns and trends, and can differ quite substantially from other subgroups (Myrskylä et al. 2013). In this section, we consider the importance of the German-speaking and Southern European country clusters in driving the convex relationship between income and fertility. To explore this, we estimate the model presented in equation (1), and include an interaction for being a German-speaking or Southern European country. The model equation is then specified as:

|  | $y_{i,c,t}=\chi_{c}*\lambda_{t}+\beta_{1}\ln\left( {inc}_{i.c, t-1} \right)+\beta_{2}{\ln\left( {inc}_{i,c,t-1} \right)}^{2}+[\beta_{3}\ln\left( {inc}_{i.c, t-1} \right)+\beta_{4}{\ln\left( {inc}_{i,c,t-1} \right)}^{2}]*\xi_{l}+\varepsilon_{i,c,t}$ | (OA2) |
| --- | --- | --- |

where $y_{i,c,t}$ is the outcome of interest (either TFR or the tempo-adjusted TFR) and $\xi_{l}$ is an indicator for whether the country is German speaking (Austria and Germany) or part of Southern Europe (Greece, Italy, Portugal, and Spain). Estimates from this model using TFR as the outcome variable are presented in Table OA4A, and those using tempo-adjusted fertility are shown in Table OA4B. Because we consider eastern Germany part of Eastern Europe, the interaction effects for Eastern Europe are identified off these eastern German regions. As such, it is not surprising that the coefficient terms on these interactions for Eastern Europe are not statistically significant, independent of whether the period or tempo-adjusted TFR is the outcome variable. Also common across Tables OA4A and OA4B is the distinctiveness of western Germany and Austria. From the coefficients for the Western European sample in column 2, the coefficient on the linear interaction term is positive and the coefficient on its square negative. One interpretation of this is that German-speaking countries are perhaps lagging behind in the emergence of a convex relationship, which might be linked to their status as laggards in the implementation of work-family reconciliation policies. However, as was the case with the subset of financial crisis years, the sample size is small and the results are likely underpowered. In addition, other factors might contribute to these outcomes. Both in Austria and western Germany, some highly developed cities form their own regions (Vienna, Hamburg, and Bremen). Furthermore, in western Germany, the population over-count in highly developed areas during the long intercensal period between 1987 and 2011 decreases the likeliness that we observe a convex relationship (see also comments in the data appendix). In any case, the effects for the German-speaking countries are not large enough to significantly impact the coefficients for the overall sample. The “distinctiveness” of the Southern European countries is less pronounced than the results for the German-speaking countries. Although the coefficients on the interaction terms for Western Europe are statistically significant when using TFR as the dependent variable, this disappears when using tempo-adjusted TFR. The outcome suggests that postponement is to some extent driving the “uniqueness” of the Southern European countries. Most importantly, the results for the coefficients that do not include the German-speaking and Southern European countries are all qualitatively the same.

**Controlling for spatial autocorrelation**

If regions in close vicinity are more similar in fertility or economic conditions compared to regions further apart in ways not completely accounted for by our models, this positive spatial autocorrelation can introduce bias in our estimates. There could, for example, be small-scale spatial dependence processes resulting from social interaction between regions (e.g. fertility in one region affecting fertility in a neighbouring region). Positive spatial autocorrelation among the model residuals indicates that the assumption of studying (conditionally) independent observations with our regression models might be violated. In such a case, our estimates of total and explained variance in the relationship between income and fertility could be inflated. This in turn implies that the obtained confidence intervals may be too narrow and the derived significance levels too high. We do not believe that these issues are of big concern in our models, as we perform our analysis at a quite high level of spatial aggregation. In addition, our country-by-year fixed effects should control for substantial parts of the existing large-scale spatial heterogeneity across European countries. Nevertheless, we explore here whether our findings from the main models are robust to models accounting for potentially uncontrolled spatial autocorrelation.

To explore whether potential bias due to spatial autocorrelation could affect findings from our main models, we estimate spatial panel models using the STATA command XSMLE (Belotti et al. 2016; see also Vitali and Billari 2015). These models come with a number of restrictions. They require a strongly balanced panel, so we adjust our sample accordingly. For our models with the TFR as the dependent variable, this necessitates cutting the sample to the years 1992–2010 and removing Romania and the Slovak Republic (for both countries the time series starts only later).^[[1]](#footnote-2)^ When using the tempo-adjusted TFR as the dependent variable, its reliance on fertility lags requires a sample limited to the period between 1994 and 2010. In addition, the models do not allow the integration of temporal lags; so for these consistency checks we use current year income and income squared as the independent variables.

In order to test whether our coefficient estimates are affected by spatial autocorrelation, we would ideally prefer to add a spatial error specification to our country-by-year fixed effects panel models. This specification isolates the spatially clustered part of the error term and generates estimates for our income variables not biased by this unobserved spatial autocorrelation. In addition, it might be interesting to explore whether there is evidence for the above-discussed small-scale spatial dependence due to social interaction. The introduction of a spatially-lagged value of the dependent variable can account for this. XSMLE allows specification of spatial error panel models, but these face problems with our datasets so that only few models converge. Thus, we specify an alternative spatial panel model that includes controls for the spatially clustered error term and for small-scale spatial dependence. However, these models require the inclusion of regional fixed effects (see Belotti et al. 2016). As such, estimates from them are not directly comparable to those shown in Tables 1 and 2 in the main text.

We present for each combination of dependent variables (TFR, tempo-adjusted TFR) and set of countries (Europe (combined), Western Europe, and Eastern Europe) a total of four or five models. The latter depends on whether we were able to obtain estimates for the spatial error panel model. The first model is a country-by-year fixed effects model analogous to Tables 1 and 2 in the main text, but using the adjusted sample described above, and current year income and income squared as the independent variables. The second model includes country-by-year fixed effects and regional fixed effects (analogous to Table OA1 in the appendix and comparable to the spatial panel model estimates). As an aside, given the potential data quality issues associated with the Romanian fertility rates, it is worthwhile to consider whether omitting Romania and the Slovak Republic in the first two models affects the results for the combined European sample and for Eastern Europe. The third model is a spatial panel model with regional fixed effects, while the fourth is a similar model that includes country-by-year fixed effects. The third model is the only model in this consistency check that does not include country-by-year fixed effects. We use it as a comparison with the fourth model to assess whether the introduction of the country-by-year fixed effects can account for any remaining unaccounted spatial autocorrelation. The fifth model, which is only presented if we were able to estimate it with STATA, is a spatial error panel model with country-by-year fixed effects.

The spatial panel model with regional fixed effects but no country-by-year fixed effects is specified as follows:

|  | $y_{i,t}=\lambda_{i}+\rho\sum_{j} w_{ij}y_{j,t}+\beta_{1}\ln\left( {inc}_{i, t} \right)+\beta_{2}{\ln\left( {inc}_{i,t} \right)}^{2}+\delta\sum_{j} w_{ij}\nu_{j,t}+\varepsilon_{i,t}$ | (OA3) |
| --- | --- | --- |

where $w_{ij}$ represents the row-standardised weight matrix that provides information on the neighbouring regions $j$ of a region $i$, and $\rho$ is the coefficient for the spatially-lagged dependent variable $y$. The error term has been split up into the spatially clustered ($\nu$) and the independent part ($\varepsilon$). For the former a coefficient δ is estimated (in the spatial econometrics literature usually denoted as$\lambda$). The spatial panel model with additional country-by-year fixed effects has the following specification:

|  | $y_{i,c,t}=\chi_{c}*\lambda_{t}+\lambda_{i}+\rho\sum_{j} w_{ij}y_{j,c,t}+\beta_{1}\ln\left( {inc}_{i,c, t} \right)+\beta_{2}{\ln\left( {inc}_{i,c,t} \right)}^{2}+\delta\sum_{j} w_{ij}\nu_{j,c,t}+\varepsilon_{i,c,t}$ | (OA4) |
| --- | --- | --- |

Finally, the spatial error panel model with country-by-year fixed effects is specified as follows:

|  | $y_{i,c,t}=\chi_{c}*\lambda_{t}+\beta_{1}\ln\left( {inc}_{i,c, t} \right)+\beta_{2}{\ln\left( {inc}_{i,c,t} \right)}^{2}+\delta\sum_{j} w_{ij}\nu_{j,c,t}+\varepsilon_{i,c,t}$ | (OA5) |
| --- | --- | --- |

In deriving our spatial weight matrix $w_{ij}$ used to capture spatial autocorrelation, we generally consider all regions that share at least one common border point as neighbours. This is also referred to as a first-order queen definition of adjacency (FOQ). In line with our country fixed effects framework, however, neighbouring regions divided by a national border are not considered neighbours. The motivation for this is that neighbouring regions *j* in another country might differ quite strongly from a region *i*, as policy and cultural conditions might be very different in that other country. Including these regions in our set of neighbouring regions *j* would thus involve the risk that we underestimate the similarity of a region *i* with its neighbouring regions *j*. By not considering neighbouring regions in other countries, we hope to better capture potential spatial autocorrelation. We also had to decide how to treat island regions with no contiguous neighbouring regions in the same country. This included Northern Ireland, the Baleares, Corse, Sardinia, Sicily, and four Greek regions in the Aegean Sea. Here we chose not to treat these regions as completely isolated, but to establish a reciprocal link between them and the nearest neighbouring region in the same country (e.g., between Sicily and Calabria).^[[2]](#footnote-3)^ This modified FOQ-weight matrix we refer to as FOQadj (adjusted for national boundaries and islands). We also specified alternative weight matrices to explore whether our outcomes differ strongly if other spatial weight matrices are considered. This included a FOQ-matrix in which also contiguous regions that are divided by a national border are considered neighbours, and matrices that use instead a three nearest neighbours specification (with one only taking nearest neighbouring regions within the same country into account, and the other considering the nearest neighbouring regions independent of the country they belong to). Estimates were robust to these sensitivity checks, suggesting that the choice of the spatial weight matrix does not strongly affect our spatial panel model outcomes.

Table OA5A presents the estimates for the models that use period TFR as the dependent variable, while Table OA5B shows those using the tempo-adjusted TFR. Before we look into the spatial models, we first explore to what degree the reduction to a balanced sample and the exclusion of Romania and the Slovak Republic affects our estimates. As expected, substantial differences are only visible in the models on Eastern Europe, where the estimates for our income variables are attenuated. This is particularly true for the model using the tempo-adjusted TFR. The outcome is in line with our assertion that the potential measurement bias in the Romanian data tends to increase the likeliness that we identify a convex relationship. However, all income estimates remain significant in the expected direction.

The results for the TFR presented in Table OA5A are generally robust to the inclusion of adjustments for spatial autocorrelation. If we contrast the second and fourth model for each of the three groups of European countries considered, the estimates for our income variables are slightly attenuated. However, they remain significant at the 5 percent level. In addition, the comparison of the third and fourth model for each group of countries demonstrates that unobserved spatial autocorrelation is substantially reduced if the country-by-year fixed effects are included.

For the models using tempo-adjusted fertility in table OA5B, balancing the panel and restricting the sample results in estimates from the models with regional fixed effects and country-by-year fixed effects that are all in the expected direction, but not statistically significant. This differs from the regional fixed effects models for the full sample shown in Table OA1 where there was only at one occasion an estimate not significant at the 10 percent level. However, the models with additional controls for spatial autocorrelation in column 4 of the different regional divisions do not provide indications that spatial autocorrelation substantially affects the models as we obtain neither for the rho nor for the delta significant outcomes. When we contrast the models in columns three and four, we see, similar as for the total fertility rate, that without country-by-year fixed effects the models would exhibit high amounts of unobserved spatial heterogeneity. For both Western and Eastern Europe, we are also able to calculate spatial error panel models. These show the robustness of the Western European results. The coefficients in model B5 are attenuated in comparison to model B1, but remain significant at the 5 percent level, while the delta coefficient for spatial autocorrelation in the error term is not significant. In the model for Eastern Europe, the coefficients in the spatial error panel model C5 are actually bigger than in model C1, but no longer significant. However, the coefficient for the error term is also negative and not significant. That the coefficient for the error term is negative suggests that in model C1 unobserved negative spatial autocorrelation is tending to decrease significance levels. Thus, model C5 also does not give a strong indication that the significant estimates for our income variables in our non-spatial model C1 are an artefact of spatial autocorrelation. Overall, these robustness checks confirm our assertion that unobserved spatial autocorrelation is unlikely to have a large effect on the key qualitative findings derived from our main models.

**References**

Belotti, F., Hughes, G., & Mortari, A. P. (2016). Spatial panel data models using Stata. Rome: University of Rome “Tor Vergata”. CEIS Tor Vergata Research Paper Series Vol. 14, Issue 5, No. 373.

Myrskylä, M., Goldstein, J. R., & Cheng, Y.-H. A. (2013). New cohort fertility forecasts for the developed world: Rises, falls and reversals. *Population and Development Review*, *39*(1), 31–56. <https://doi.org/10.1111/j.1728-4457.2013.00572.x>.

Vitali, A., & Billari, F. C. (2017). Changing determinants of low fertility and diffusion: A spatial analysis for Italy. *Population, Space and Place*, *23*(2), e1998. <https://doi.org/10.1002/psp.1998>.

**Table OA1** Country-by-year and regional fixed effects

|  | Total fertility rate | | | Tempo-adjusted  total fertility rate | | |
| --- | --- | --- | --- | --- | --- | --- |
|  | Europe (combined sample) | Western Europe | Eastern Europe | Europe (combined sample) | Western Europe | Eastern Europe |
| Independent variables | (1) | (2) | (3) | (4) | (5) | (6) |
| Prior year compensation per capita (natural log) | -1.233  (0.539)* | -2.012  (1.053)^+^ | -2.448  (0.724)** | -0.785  (0.465)^+^ | -1.983  (0.843)* | -1.907  (0.979)^+^ |
| Prior year compensation per capita squared (natural log) | 0.075  (0.031)* | 0.106  (0.056)^+^ | 0.168  (0.045)** | 0.042  (0.027) | 0.092  (0.042)* | 0.131  (0.062)* |
| Constant | 6.444  (2.350)** | 11.196  (4.907)* | 10.322  (2.885)** | 5.465  (2.090)** | 12.245  (4.235)** | 8.619  (3.834)* |
|  |  |  |  |  |  |  |
| Inversion point (in 2005 Euros) | 3463 € | 13,758 € | 1487 € | 11,988 € | 46,502 € | 1417 € |
| Regional fixed effects | Yes | Yes | Yes | Yes | Yes | Yes |
| Country-year interacted fixed effects | Yes | Yes | Yes | Yes | Yes | Yes |
|  |  |  |  |  |  |  |
| Number of observations | 5703 | 4522 | 1181 | 5205 | 4116 | 1089 |
| Regions | 256 | 200 | 56 | 256 | 200 | 56 |
| Adjusted R-squared | 0.834 | 0.743 | 0.943 | 0.712 | 0.651 | 0.854 |

Notes: Robust standard errors in parentheses; ** p<0.01, * p<0.05, ^+^ p<0.1

Employee compensation is defined as the total remuneration, in cash or in kind, payable by an employer to an employee in return for work done by the latter. It consists of wages and salaries, and of employers' social contributions and is adjusted to 2005 Euros. Tempo-adjusted TFR is the adjusted total fertility rate, which is equal to TFR/(1-∆MACB), where ∆MACB is the annual change in the mean age at childbearing. Inversion points are calculated using coefficients rounded to the eighth decimal point.

Source: Eurostat, Statistical Offices, Cambridge Econometrics; own calculations

**Table OA2** Gross Domestic Product per capita as measure of economic development

|  | Total fertility rate | | | Tempo-adjusted  total fertility rate | | |
| --- | --- | --- | --- | --- | --- | --- |
|  | Europe (combined sample) | Western Europe | Eastern Europe | Europe (combined sample) | Western Europe | Eastern Europe |
| Independent variables | (1) | (2) | (3) | (4) | (5) | (6) |
| Prior year GDP per capita (natural log) | -1.551  (0.116)** | -0.711  (0.302)* | -3.119  (0.246)** | -1.307  (0.121)** | -2.367  (0.346)** | -2.336  (0.286)** |
| Prior year GDP per capita squared (natural log) | 0.073  (0.006)** | 0.032  (0.014)* | 0.163  (0.013)** | 0.063  (0.006)** | 0.114  (0.017)** | 0.124  (0.016)** |
| Constant | 9.696  (0.570)** | 5.470  (1.529)** | 16.146  (1.106)** | 8.800  (0.601)** | 13.992  (1.760)** | 12.337  (1.268)** |
|  |  |  |  |  |  |  |
| Inversion point (in 2005 Euros) | 38,729 € | 67,380 € | 14,367 € | 29,866 € | 30,860 € | 12,062 € |
| Regional fixed effects | No | No | No | No | No | No |
| Country-year interacted fixed effects | Yes | Yes | Yes | Yes | Yes | Yes |
|  |  |  |  |  |  |  |
| Number of observations | 5706 | 4525 | 1181 | 5208 | 4119 | 1089 |
| Regions | 256 | 200 | 56 | 256 | 200 | 56 |
| Adjusted R-squared | 0.825 | 0.804 | 0.839 | 0.768 | 0.760 | 0.764 |

Notes: Robust standard errors in parentheses; ** p<0.01, * p<0.05, ^+^ p<0.1

GDP is the Gross Domestic Product, defined as Total Gross Value Added plus taxes less subsidies on products. It is adjusted to 2005 Euros. Tempo-adjusted TFR is the adjusted total fertility rate, which is equal to TFR/(1-∆MACB), where ∆MACB is the annual change in the mean age at childbearing. Inversion points are calculated using coefficients rounded to the eighth decimal point.

Source: Eurostat, Statistical Offices, Cambridge Econometrics; own calculations

**Table OA3A** Estimates for the periods before and after the onset of the 2007 financial crisis, total fertility rate as dependent variable

|  | 1990-2007 | | | 2008-2012 | | |
| --- | --- | --- | --- | --- | --- | --- |
|  | Europe (combined sample) | Western Europe | Eastern Europe | Europe (combined sample) | Western Europe | Eastern Europe |
| Independent variables | (1) | (2) | (3) | (4) | (5) | (6) |
| Prior year compensation per capita (natural log) | -1.581  (0.098)** | -1.465  (0.230)** | -2.337  (0.231)** | -0.355  (0.194)^+^ | 2.812  (0.462)** | -1.302  (0.303)** |
| Prior year compensation per capita squared (natural log) | 0.080  (0.005)** | 0.074  (0.012)** | 0.130  (0.014)** | 0.018  (0.011)^+^ | -0.145  (0.023)** | 0.075  (0.018)** |
| Constant | 9.238  (0.444)** | 9.156  (1.093)** | 11.683  (0.928)** | 3.687  (0.869)** | -11.679  (2.249)** | 6.957  (1.239)** |
|  |  |  |  |  |  |  |
| Inversion point (in 2005 Euros) | 18,635 € | 19,887 € | 8138 € | 16,824 € | 16,403 € | 5869 € |
| Regional fixed effects | No | No | No | No | No | No |
| Country-year interacted fixed effects | Yes | Yes | Yes | Yes | Yes | Yes |
|  |  |  |  |  |  |  |
| Number of observations | 4426 | 3525 | 901 | 1277 | 997 | 280 |
| Regions | 256 | 200 | 56 | 256 | 200 | 56 |
| Adjusted R-squared | 0.819 | 0.788 | 0.865 | 0.844 | 0.850 | 0.539 |

Notes: Robust standard errors in parentheses; ** p<0.01, * p<0.05, ^+^ p<0.1

Employee compensation is defined as the total remuneration, in cash or in kind, payable by an employer to an employee in return for work done by the latter. It consists of wages and salaries, and of employers' social contributions and is adjusted to 2005 Euros. Inversion points are calculated using coefficients rounded to the eighth decimal point.

Source: Eurostat, Statistical Offices, Cambridge Econometrics; own calculations

**Table OA3B** Estimates for the periods before and after the onset of the 2007 financial crisis, tempo-adjusted total fertility rate as dependent variable

|  | 1990-2007 | | | 2008-2012 | | |
| --- | --- | --- | --- | --- | --- | --- |
|  | Europe (combined sample) | Western Europe | Eastern Europe | Europe (combined sample) | Western Europe | Eastern Europe |
| Independent variables | (1) | (2) | (3) | (4) | (5) | (6) |
| Prior year compensation per capita (natural log) | -1.355  (0.116)** | -2.563  (0.279)** | -1.727  (0.271)** | -0.530  (0.193)^+^ | 0.924  (0.641) | -1.165  (0.395)** |
| Prior year compensation per capita squared (natural log) | 0.070  (0.006)** | 0.133  (0.014)** | 0.098  (0.017)** | 0.032  (0.011)^+^ | -0.043  (0.033) | 0.071  (0.024)** |
| Constant | 8.610  (0.539)** | 14.055  (1.314)** | 9.168  (1.079)** | 3.898  (0.856)** | -2.808  (3.103)** | 6.176  (1.596)** |
|  |  |  |  |  |  |  |
| Inversion point (in 2005 Euros) | 15,772 € | 15,130 € | 7005 € | 4030 € | 45,064 € | 3802 € |
| Regional fixed effects | No | No | No | No | No | No |
| Country-year interacted fixed effects | Yes | Yes | Yes | Yes | Yes | Yes |
|  |  |  |  |  |  |  |
| Number of observations | 3934 | 3125 | 809 | 1271 | 991 | 280 |
| Regions | 256 | 200 | 56 | 256 | 200 | 56 |
| Adjusted R-squared | 0.764 | 0.758 | 0.780 | 0.778 | 0.755 | 0.724 |

Notes: Robust standard errors in parentheses; ** p<0.01, * p<0.05, ^+^ p<0.1

Employee compensation is defined as the total remuneration, in cash or in kind, payable by an employer to an employee in return for work done by the latter. It consists of wages and salaries, and of employers' social contributions and is adjusted to 2005 Euros. Tempo-adjusted TFR is the adjusted total fertility rate, which is equal to TFR/(1-∆MACB), where ∆MACB is the annual change in the mean age at childbearing. Inversion points are calculated using coefficients rounded to the eighth decimal point.

Source: Eurostat, Statistical Offices, Cambridge Econometrics; own calculations

**Table OA4A** Controlling for belonging to a German-speaking or Southern European country, total fertility rate as dependent variable

|  | Interaction (German-speaking countries) | | | Interaction (Southern Europe) | | |
| --- | --- | --- | --- | --- | --- | --- |
|  | Europe (combined sample) | Western Europe | Eastern Europe | Europe (combined sample) | Western Europe | Eastern Europe |
| Independent variables | (1) | (2) | (3) | (4) | (5) | (6) |
| Prior year comp. p. cp. (nat. log) [1] | -1.309  (0.093)** | -1.023  (0.231)** | -2.285  (0.188)** | -1.225  (0.090)** | -1.196  (0.447)** | -2.271  (0.183)** |
| Prior year comp. p. cp. squared (nat. log) [2] | 0.067  (0.005)** | 0.052  (0.012)** | 0.130  (0.011)** | 0.061  (0.005)** | 0.059  (0.023)* | 0.129  (0.011)** |
| [1] * German-speaking | 8.508  (1.210)** | 9.848  (1.383)** | -3.319  (2.253) |  |  |  |
| [2] * German-speaking | -0.448  (0.063)** | -0.518  (0.072)** | 0.177 (0.126) |  |  |  |
| [1] * Southern Europe |  |  |  | -2.372  (0.489)** | -2.401  (0.655)** |  |
| [2] * Southern Europe |  |  |  | 0.137  (0.027)** | 0.139  (0.035)** |  |
| Constant | -32.527  (5.792)** | 6.581  (1.078)** | 11.313  (0.763)** | 8.079  (0.420)** | 17.696  (2.124)** | 11.230  (0.745)** |
|  |  |  |  |  |  |  |
| Inversion point (in 2005 Euros) | 17,388 € | 20,300 € | 6624 € | 22,220 € | 25,051 € | 6680 € |
| Regional fixed effects | No | No | No | No | No | No |
| Country-year interacted fixed effects | Yes | Yes | Yes | Yes | Yes | Yes |
|  |  |  |  |  |  |  |
| Number of observations | 5703 | 4522 | 1181 | 5703 | 4522 | 1181 |
| Regions | 256 | 200 | 56 | 256 | 200 | 56 |
| Adjusted R-squared | 0.826 | 0.806 | 0.834 | 0.826 | 0.806 | 0.835 |

Notes: Robust standard errors in parentheses; ** p<0.01, * p<0.05, ^+^ p<0.1

Employee compensation is defined as the total remuneration, in cash or in kind, payable by an employer to an employee in return for work done by the latter. It consists of wages and salaries, and of employers' social contributions and is adjusted to 2005 Euros. “German speaking” includes the countries Germany and Austria; Switzerland is not part of the sample. As such, the interaction effects for Eastern Europe are identified solely off of eastern German regions. “Southern Europe” includes the countries Greece, Italy, Portugal, and Spain. None of the Southern European countries are considered “Eastern European”; so the interacted terms are not identified for this sample and the income coefficients are equivalent to those in Table 1. Inversion points are calculated using coefficients rounded to the eighth decimal point.

Source: Eurostat, Statistical Offices, Cambridge Econometrics; own calculations

**Table OA4B** Controlling for belonging to a German-speaking or Southern European country, tempo-adjusted total fertility rate as dependent variable

|  | Interaction (German-speaking countries) | | | Interaction (Southern Europe) | | |
| --- | --- | --- | --- | --- | --- | --- |
|  | Europe (combined sample) | Western Europe | Eastern Europe | Europe (combined sample) | Western Europe | Eastern Europe |
| Independent variables | (1) | (2) | (3) | (4) | (5) | (6) |
| Prior year comp. p. cp. (nat. log) [1] | -1.152  (0.099)** | -2.205  (0.268)** | -1.678  (0.225)** | -1.047  (0.096)** | -2.222  (0.503)** | -1.754  (0.221)** |
| Prior year comp. p. cp. squared (nat. log) [2] | 0.061  (0.005)** | 0.115  (0.014)** | 0.097  (0.014)** | 0.056  (0.005)** | 0.116  (0.026)** | 0.102  (0.013)** |
| [1] * German-speaking | 8.371  (1.494)** | 9.008  (1.659)** | -11.516  (8.019) |  |  |  |
| [2] * German-speaking | -0.439  (0.078)** | -0.472  (0.086)** | 0.648 (0.434) |  |  |  |
| [1] * Southern Europe |  |  |  | -1.897  (0.675)** | -0.722  (0.834) |  |
| [2] * Southern Europe |  |  |  | 0.102  (0.037)** | 0.042  (0.046) |  |
| Constant | 7.266  (0.461)** | -30.706  (7.850)** | 8.586  (0.901)** | 6.961  (0.446)** | 15.258  (2.966)** | 8.875  (0.884)** |
|  |  |  |  |  |  |  |
| Inversion point (in 2005 Euros) | 12,676 € | 14,046 € | 5578 € | 11,507 € | 14,754 € | 5324 € |
| Regional fixed effects | No | No | No | No | No | No |
| Country-year interacted fixed effects | Yes | Yes | Yes | Yes | Yes | Yes |
|  |  |  |  |  |  |  |
| Number of observations | 5205 | 4116 | 1089 | 5205 | 4116 | 1089 |
| Regions | 256 | 200 | 56 | 256 | 200 | 56 |
| Adjusted R-squared | 0.770 | 0.763 | 0.765 | 0.770 | 0.762 | 0.764 |

Notes: Robust standard errors in parentheses; ** p<0.01, * p<0.05, ^+^ p<0.1

Employee compensation is defined as the total remuneration, in cash or in kind, payable by an employer to an employee in return for work done by the latter. It consists of wages and salaries, and of employers' social contributions and is adjusted to 2005 Euros. Tempo-adjusted TFR is the adjusted total fertility rate, which is equal to TFR/(1-∆MACB), where ∆MACB is the annual change in the mean age at childbearing. “German speaking” includes the countries Germany and Austria; Switzerland is not part of the sample. As such, the interaction effects for Eastern Europe are identified solely off of eastern German regions. “Southern Europe” includes the countries Greece, Italy, Portugal, and Spain. None of the Southern European countries are considered “Eastern European”; so the interacted terms are not identified for this sample and the income coefficients are equivalent to those in Table 1. Inversion points are calculated using coefficients rounded to the eighth decimal point.

Source: Eurostat, Statistical Offices, Cambridge Econometrics; own calculations

**Table OA5A** Spatial panel model specifications accounting for unobserved spatial autocorrelation, total fertility rate as dependent variable

|  | Europe (combined) | | | |
| --- | --- | --- | --- | --- |
|  | Country-by-year (CBY) fixed effects only | CBY and regional fixed effects | Regional fixed effects and spatial effects (lag and error) | CBY and regional fixed effects, and spatial effects (lag and error) |
| Independent variables | (A1) | (A2) | (A3) | (A4) |
| Prior year comp. p. cp. (nat. log) | -1.080  (0.093)** | -1.387  (0.574)* | -1.580  (0.556)** | -1.343  (0.548)* |
| Prior year comp. p. cp. squared (nat. log) | 0.055  (0.005)** | 0.077  (0.032)** | 0.099  (0.032)** | 0.077  (0.031)* |
| Rho |  |  | -0.454  (0.067)** | 0.090  (0.059) |
| Delta |  |  | 0.863  (0.019)** | 0.319  (0.102)** |
|  |  |  |  |  |
| Constant | 6.998  (0.424)** | 7.751  (2.565)** |  |  |
|  |  |  |  |  |
| Inversion point (in 2005 Euros) | 19,973 € | 7858 € | 2886 € | 5977 € |
| Regional fixed effects | No | Yes | Yes | Yes |
| Country-year interacted fixed effects | Yes | Yes | No | Yes |
| Spatial weight matrix | No | No | FOQadj | FOQadj |
|  |  |  |  |  |
| Number of observations | 4636 | 4636 | 4636 | 4636 |
| Regions | 244 | 244 | 244 | 244 |

Notes at end of table

|  | Western Europe | | | |
| --- | --- | --- | --- | --- |
|  | Country-by-year (CBY) fixed effects only | CBY and regional fixed effects | Regional fixed effects and spatial effects (lag and error) | CBY and regional fixed effects, and spatial effects (lag and error) |
| Independent variables | (B1) | (B2) | (B3) | (B4) |
| Prior year comp. p. cp. (nat. log) | -0.864  (0.237)** | -2.062  (0.993)* | -1.513  (0.609)* | -1.977  (0.979)* |
| Prior year comp. p. cp. squared (nat. log) | 0.043  (0.012)** | 0.106  (0.053)* | 0.090  (0.034)** | 0.105  (0.053)* |
| Rho |  |  | -0.472  (0.069)** | -0.090  (0.225) |
| Delta |  |  | 0.877  (0.017)** | 0.463  (0.233)* |
|  |  |  |  |  |
| Constant | 5.971  (1.125)** | 11.573  (4.679)* |  |  |
|  |  |  |  |  |
| Inversion point (in 2005 Euros) | 22,091 € | 16,018 € | 4364 € | 12,128 € |
| Regional fixed effects | No | Yes | Yes | Yes |
| Country-year interacted fixed effects | Yes | Yes | No | Yes |
| Spatial weight matrix | No | No | FOQadj | FOQadj |
|  |  |  |  |  |
| Number of observations | 3800 | 3800 | 3800 | 3800 |
| Regions | 200 | 200 | 200 | 200 |

Notes at end of table

|  | Eastern Europe | | | | |
| --- | --- | --- | --- | --- | --- |
|  | Country-by-year (CBY) fixed effects only | CBY and regional fixed effects | Regional fixed effects and spatial effects (lag and error) | CBY and regional fixed effects, and spatial effects (lag and error) | CBY fixed effects and spatial effects (error) |
| Independent variables | (C1) | (C2) | (C3) | (C4) | (C5) |
| Prior year comp. p. cp. (nat. log) | -1.357  (0.153)** | -2.153  (0.847)* | -1.179  (0.602)^+^ | -1.957  (0.895)* | -1.833  (0.789)* |
| Prior year comp. p. cp. squared (nat. log) | 0.072  (0.009)** | 0.140  (0.050)** | 0.083  (0.037)* | 0.129  (0.054)* | 0.119  (0.047)* |
| Rho |  |  | -0.423  (0.166)* | 0.140  (0.132) |  |
| Delta |  |  | 0.955  (0.010)** | 0.141  (0.129) | 0.257  (0.131)* |
|  |  |  |  |  |  |
| Constant | 7.528  (0.615)** | 9.890  (3.500)** |  |  |  |
|  |  |  |  |  |  |
| Inversion point (in 2005 Euros) | 12,790 € | 2231 € | 1259 € | 1987 € | 2204 € |
| Regional fixed effects | No | Yes | Yes | Yes | No |
| Country-year interacted fixed effects | Yes | Yes | No | Yes | Yes |
| Spatial weight matrix | No | No | FOQadj | FOQadj | FOQadj |
|  |  |  |  |  |  |
| Number of observations | 836 | 836 | 836 | 836 | 836 |
| Regions | 44 | 44 | 44 | 44 | 44 |

Notes: Robust standard errors in parentheses; ** p<0.01, * p<0.05, ^+^ p<0.1

Employee compensation is defined as the total remuneration, in cash or in kind, payable by an employer to an employee in return for work done by the latter. It consists of wages and salaries, and of employers' social contributions and is adjusted to 2005 Euros. The country-by-year fixed effects models are analogous to estimates presented in Table 1, but are calculated for the balanced panel and using same year income variables. Rho represents the estimate for the spatially-lagged value of the dependent variable, while delta gives the estimate for the spatially autocorrelated part of the error. If these estimates are significant, this is indicative of spatial autocorrelation not accounted for by our models. Spatial panel models (A3, A4, B3, B4, C3-C5) are estimated using the XSMLE command in STATA applying a spatial weight matrix that generally considers all regions that share at least one common border point as neighbours (first-order queen, FOQ). This weight matrix has been adjusted to only include neighbouring regions in the same country. In addition, island regions with no contiguous neighbouring regions in the same country are linked with a reciprocal link to the nearest neighbouring region in the same country (the motivation for these decisions is given in the text). This modified spatial weight matrix is referred to as FOQadj. Inversion points are calculated using coefficients rounded to the eighth decimal point.

Source: Eurostat, Statistical Offices, Cambridge Econometrics; own calculations

**Table OA5B** Spatial panel model specifications accounting for unobserved spatial autocorrelation, tempo-adjusted total fertility rate as dependent variable

|  | Europe (combined) | | | |
| --- | --- | --- | --- | --- |
|  | Country-by-year (CBY) fixed effects only | CBY and regional fixed effects | Regional fixed effects and spatial effects (lag and error) | CBY and regional fixed effects, and spatial effects (lag and error) |
| Independent variables | (A1) | (A2) | (A3) | (A4) |
| Prior year comp. p. cp. (nat. log) | -1.060  (0.110)** | -0.858  (0.578) | -0.373  (0.311) | -0.836  (0.551) |
| Prior year comp. p. cp. squared (nat. log) | 0.056  (0.006)** | 0.042  (0.032) | 0.021  (0.017) | 0.042  (0.030) |
| Rho |  |  | 0.830  (0.018)** | 0.013  (0.097) |
| Delta |  |  | -0.584  (0.049)** | 0.043  (0.129) |
|  |  |  |  |  |
| Constant | 6.517  (0.505)** | 6.083  (2.683)* |  |  |
|  |  |  |  |  |
| Inversion point (in 2005 Euros) | 12,754 € | 25,282 € | 6739 € | 22,072 € |
| Regional fixed effects | No | Yes | Yes | Yes |
| Country-year interacted fixed effects | Yes | Yes | No | Yes |
| Spatial weight matrix | No | No | FOQadj | FOQadj |
|  |  |  |  |  |
| Number of observations | 4148 | 4148 | 4148 | 4148 |
| Regions | 244 | 244 | 244 | 244 |

Notes at end of table

|  | Western Europe | | | | |
| --- | --- | --- | --- | --- | --- |
|  | Country-by-year (CBY) fixed effects only | CBY and regional fixed effects | Regional fixed effects and spatial effects (lag and error) | CBY and regional fixed effects, and spatial effects (lag and error) | CBY fixed effects and spatial effects (error) |
| Independent variables | (B1) | (B2) | (B3) | (B4) | (B5) |
| Prior year comp. p. cp. (nat. log) | -2.142  (0.278)** | -1.344  (0.972) | -1.001  (0.352)** | -1.340  (0.931) | -1.673  (0.850)* |
| Prior year comp. p. cp. squared (nat. log) | 0.112  (0.014)** | 0.064  (0.049) | 0.055  (0.019)** | 0.064  (0.047) | 0.086  (0.044)* |
| Rho |  |  | 0.811  (0.024)** | 0.040  (0.105) |  |
| Delta |  |  | -0.569  (0.060)** | -0.017  (0.134) | 0.031  (0.038) |
|  |  |  |  |  |  |
| Constant | 11.698  (1.320)** | 8.747  (4.789)^+^ |  |  |  |
|  |  |  |  |  |  |
| Inversion point (in 2005 Euros) | 13,667 € | 35,072 € | 8467 € | 34,538 € | 16,914 € |
| Regional fixed effects | No | Yes | Yes | Yes | No |
| Country-year interacted fixed effects | Yes | Yes | No | Yes | Yes |
| Spatial weight matrix | No | No | FOQadj | FOQadj | FOQadj |
|  |  |  |  |  |  |
| Number of observations | 3400 | 3400 | 3400 | 3400 | 3400 |
| Regions | 200 | 200 | 200 | 200 | 200 |

Notes at end of table

|  | Eastern Europe | | | | |
| --- | --- | --- | --- | --- | --- |
|  | Country-by-year (CBY) fixed effects only | CBY and regional fixed effects | Regional fixed effects and spatial effects (lag and error) | CBY and regional fixed effects, and spatial effects (lag and error) | CBY fixed effects and spatial effects (error) |
| Independent variables | (C1) | (C2) | (C3) | (C4) | (C5) |
| Prior year comp. p. cp. (nat. log) | -0.590  (0.212)** | -1.187  (1.150) | 0.060  (0.273) | -1.077  (1.087) | -1.162  (0.804) |
| Prior year comp. p. cp. squared (nat. log) | 0.029  (0.013)* | 0.072  (0.071) | -0.005  (0.017) | 0.065  (0.066) | 0.067  (0.051) |
| Rho |  |  | 0.927  (0.009)** | 0.029  (0.090) |  |
| Delta |  |  | -0.714  (0.074)** | -0.073  (0.104) | -0.055  (0.083) |
|  |  |  |  |  |  |
| Constant | 4.291  (0.847)** | 6.637  (4.661) |  |  |  |
|  |  |  |  |  |  |
| Inversion point (in 2005 Euros) | 26,252 € | 3729 € | 373 € | 4067 € | 5652 € |
| Regional fixed effects | No | Yes | Yes | Yes | No |
| Country-year interacted fixed effects | Yes | Yes | No | Yes | Yes |
| Spatial weight matrix | No | No | FOQadj | FOQadj | FOQadj |
|  |  |  |  |  |  |
| Number of observations | 748 | 748 | 748 | 748 | 748 |
| Regions | 44 | 44 | 44 | 44 | 44 |

Notes: Robust standard errors in parentheses; ** p<0.01, * p<0.05, ^+^ p<0.1

Employee compensation is defined as the total remuneration, in cash or in kind, payable by an employer to an employee in return for work done by the latter. It consists of wages and salaries, and of employers' social contributions and is adjusted to 2005 Euros. Tempo-adjusted TFR is the adjusted total fertility rate, which is equal to TFR/(1-∆MACB), where ∆MACB is the annual change in the mean age at childbearing. The country-by-year fixed effects models are analogous to estimates presented in Table 1, but are calculated for the balanced panel and using same year income variables. Rho represents the estimate for the spatially-lagged value of the dependent variable, while delta gives the estimate for the spatially autocorrelated part of the error. If these estimates are significant, this is indicative of spatial autocorrelation not accounted for by our models. Spatial panel models (A3, A4, B3-B5, C3-C5) are estimated using the XSMLE command in STATA applying a spatial weight matrix that generally considers all regions that share at least one common border point as neighbours (first-order queen, FOQ). This weight matrix has been adjusted to only include neighbouring regions in the same country. In addition, island regions with no contiguous neighbouring regions in the same country are linked with a reciprocal link to the nearest neighbouring region in the same country (the motivation for these decisions is given in the text). This modified spatial weight matrix is referred to as FOQadj. Inversion points are calculated using coefficients rounded to the eighth decimal point.

Source: Eurostat, Statistical Offices, Cambridge Econometrics; own calculations

1. A small number of regions in our dataset only have incomplete income information for 2011. [↑](#footnote-ref-2)
2. We determined nearest neighbours by calculating the spherical distances between the geographical centroids of the regions. [↑](#footnote-ref-3)
